# Spatial Distribution of Arctic Bacterioplankton Abundance Is Linked to Distinct Water Masses and Summertime Phytoplankton Bloom Dynamics (Fram Strait, 79°N)

**DOI:** 10.3389/fmicb.2021.658803

**Published:** 2021-05-10

**Authors:** Magda G. Cardozo-Mino, Eduard Fadeev, Verena Salman-Carvalho, Antje Boetius

**Affiliations:** ^1^Max Planck Institute for Marine Microbiology, Bremen, Germany; ^2^Alfred Wegener Institute, Helmholtz Center for Polar and Marine Research, Bremerhaven, Germany; ^3^MARUM, University of Bremen, Bremen, Germany

**Keywords:** Arctic Ocean, Fram Strait, bacterioplankton, CARD-FISH, water column

## Abstract

The Arctic is impacted by climate warming faster than any other oceanic region on Earth. Assessing the baseline of microbial communities in this rapidly changing ecosystem is vital for understanding the implications of ocean warming and sea ice retreat on ecosystem functioning. Using CARD-FISH and semi-automated counting, we quantified 14 ecologically relevant taxonomic groups of bacterioplankton (*Bacteria* and *Archaea*) from surface (0–30 m) down to deep waters (2,500 m) in summer ice-covered and ice-free regions of the Fram Strait, the main gateway for Atlantic inflow into the Arctic Ocean. Cell abundances of the bacterioplankton communities in surface waters varied from 10^5^ cells mL^–1^ in ice-covered regions to 10^6^ cells mL^–1^ in the ice-free regions. Observations suggest that these were overall driven by variations in phytoplankton bloom conditions across the Strait. The bacterial groups *Bacteroidetes* and *Gammaproteobacteria* showed several-fold higher cell abundances under late phytoplankton bloom conditions of the ice-free regions. Other taxonomic groups, such as the *Rhodobacteraceae*, revealed a distinct association of cell abundances with the surface Atlantic waters. With increasing depth (>500 m), the total cell abundances of the bacterioplankton communities decreased by up to two orders of magnitude, while largely unknown taxonomic groups (e.g., SAR324 and SAR202 clades) maintained constant cell abundances throughout the entire water column (ca. 10^3^ cells mL^–1^). This suggests that these enigmatic groups may occupy a specific ecological niche in the entire water column. Our results provide the first quantitative spatial variations assessment of bacterioplankton in the summer ice-covered and ice-free Arctic water column, and suggest that further shift toward ice-free Arctic summers with longer phytoplankton blooms can lead to major changes in the associated standing stock of the bacterioplankton communities.

## Introduction

Atmospheric and oceanic warming has a substantial impact on the Arctic Ocean already today (Dobricic et al., 2016; Sun et al., 2016; Dai et al., 2019). The strong decline in sea ice coverage (Peng and Meier, 2018; Dai et al., 2019) and heat transfer by the Atlantic water inflow (Beszczynska-Möller et al., 2012; Rudels et al., 2012; Walczowski et al., 2017) will affect stratification of the water column and can lead to an increase in upward mixing of the Atlantic core water, a process also termed “Atlantification” (Polyakov et al., 2017). The main inflow of Atlantic water into the Arctic Ocean occurs through the Fram Strait (Beszczynska-Möller et al., 2011), making it a sentinel region for observing the ongoing changes in the Arctic marine ecosystem (Soltwedel et al., 2005, 2016). The Fram Strait is also the main deep-water gateway between the Atlantic and the Arctic Ocean. It hosts two distinct hydrographic regimes; the West Spitsbergen Current (WSC) that carries relatively warm and saline Atlantic water northward along the Svalbard shelf (Beszczynska-Möller et al., 2012; von Appen et al., 2015), and the East Greenland Current (EGC) that transports cold polar water and sea ice southwards from the Arctic Ocean along the ice-covered Greenland shelf (de Steur et al., 2009; Wekerle et al., 2017).

Sea ice conditions have a strong impact on the seasonal ecological dynamics in Fram Strait and the whole Arctic Ocean (Wassmann and Reigstad, 2011), affecting light availability and stratification in the water column. The presence of sea ice and snow cover can suppress the seasonal phytoplankton bloom in the water column through light limitation (Mundy et al., 2005; Leu et al., 2011), or change its timing, e.g., by increasing stratification of the surface waters once the ice melts (Korhonen et al., 2013). Also, sea-ice algae can make up a significant proportion of the annual productivity (Leu et al., 2011; Boetius et al., 2013; Fernández-Méndez et al., 2014). Previous summer observations in the Fram Strait already suggested that total cell abundances and productivity of bacterioplankton communities in surface waters are driven by environmental parameters associated with phytoplankton bloom dynamics (Fadeev et al., 2018), such as the availability and composition of organic matter (Piontek et al., 2015; Engel et al., 2019), with differences between ice-covered and ice-free regions (Piontek et al., 2014; Fadeev et al., 2018).

Long-term summer observations in the region, conducted in the framework of the Long-Term Ecological Research site HAUSGARTEN, revealed strong ecological variations associated with the Atlantic Meridional Overturning Circulation (Soltwedel et al., 2016). Warming events during the past decades influenced seasonal phytoplankton blooms by causing a slow but continuous increase in biomass, and a shift from diatom- to flagellate-dominated communities (Nöthig et al., 2015; Engel et al., 2017; Basedow et al., 2018). It has been recently observed that phytoplankton blooms show an increasing partitioning of the produced organic carbon into the dissolved phase (Engel et al., 2019), which may result in a more active microbial loop in the upper ocean and less export of particulate matter (Vernet et al., 2017; Fadeev et al., 2020). In times of a rapidly changing Arctic ecosystem, investigating structure and dynamics of bacterioplankton communities remains a key component to the understanding of current changes in this environment. However, so far, an assessment of associated responses of the key bacterial taxa responsible for an increased recycling is missing, especially with regard to shifts in standing stocks.

To date, the majority of Arctic bacterioplankton studies are performed using high-throughput sequencing of the 16S rRNA gene, which cannot be directly converted to absolute standing stock abundances of specific taxonomic groups due to polymerase chain reaction (PCR) primers selection (Fadeev et al., 2021), as well as other quantitative biases (Gloor et al., 2017; Kumar et al., 2017; Piwosz et al., 2020). Here we used semi-automatic CAtalyzed Reporter Deposition-Fluorescence *In Situ* Hybridization (CARD-FISH; Pernthaler et al., 2002). The power of this technique lies in the ability to acquire absolute abundance of the targeted taxonomic groups free of compositional effect (Amann et al., 1990). Besides the ability to target and quantify specific taxonomic groups, the retrieval of a positive hybridization signal furthermore indicates that the analyzed cell was alive and active before fixation (Amann et al., 1990; DeLong et al., 1999). Automatization of the microscopic examination and counting procedure can reach a high-throughput standard (Schattenhofer et al., 2009; Teeling et al., 2012; Bižić-Ionescu et al., 2015; Bennke et al., 2016).

Using CARD-FISH and semi-automated cell counting, we quantified bacterial and archaeal cell abundances of 12 taxonomic groups selected based on a 16S rRNA gene survey of water column microbial communities during the same summer expedition in the Fram Strait (Fadeev et al., 2020) (Supplementary Table 1). Samples were collected from 11 stations at four different depths, targeting previously defined layers of the water column in the Fram Strait (Rudels et al., 2012): surface mixed layer (0–30 m; seasonally mixed layer of Atlantic and Arctic waters), epipelagic (100 m; mainly modified Atlantic water), deep mesopelagic (500–1,000 m; intermediate water), and bathypelagic (1,200–2,500 m; Eurasian Basin deep waters; Table 1). The main objective of this study was to assess the standing stocks of key taxonomic groups in the summer bacterioplankton across the Fram Strait. Using high-throughput cell counts data of bacterioplankton cell abundances we tested the following hypotheses: (1) in surface waters, the abundances of different bacterioplankton taxonomic groups are associated with phytoplankton bloom conditions, and, are linked to the abundances of specific phytoplankton populations; (2) water depth structures the bacterioplankton communities, and (3) differences between communities in ice-covered and ice-free regions decrease with increasing water depth.

**TABLE 1:** Environmental parameters measured at different stations and microscopy counts (cells mL^–1^) of diatoms and *Phaeocystis* spp.

**Region**	**Station**	**PANGAEA Event ID**	**Water layer**	**Lat (°N)**	**Lon (°E)**	**Depth (m)**	**Temp (°C)**	**Sal**	**Chl a (μg L^–1^)**	**NO_3_ (μmol L^–1^)**	Δ**NO_3_ (μmol L^–1^)**	**PO_4_ (μmol L^–1^)**	Δ**PO_4_ (μmol L^–1^)**	**N:P**	**NH_4_ (μmol L^–1^)**	Δ**NH_4_ (μmol L^–1^)**	**SiO_3_ (μmol L^–1^)**	Δ**SiO_3_ (μmol L^–1^)**	**Diatoms (pennate and centric)**	***Phaeocystis* spp.**
EGC	EG1	PS99/051-2	SUR	78.99	−5.42	13	−1.51	33.17	1.66	3.75 ± 0.01	3.55 ± 0.01	0.46 ± 0.00	0.13 ± 0.00	8.15	0 ± 0.00	0.00	4.50 ± 0.00	0.14 ± 0.02	148	163
EGC	EG1	PS99/051-2	EPI	78.99	−5.42	100	−1.11	34.23		8.44 ± 0.04		0.63 ± 0.00		13.40	0 ± 0.01		4.04 ± 0.00			
EGC	EG1	PS99/051-2	MES	78.99	−5.42	971	−0.14	34.89		12.62 ± 0.03		0.85 ± 0.00		14.85	0 ± 0.03		7.00 ± 0.04			
EGC	EG4	PS99/048-11	SUR	78.82	−2.73	24	−1.17	34.48	1.52	4.92 ± 0.02	6.34 ± 0.01	0.50 ± 0.00	0.35 ± 0.00	9.84	0 ± 0.02	0.08 ± 0.01	2.44 ± 0.14	2.38 ± 0.34	65	13178
EGC	EG4	PS99/048-11	EPI	78.82	−2.73	100	3.86	35.05		10.97 ± 0.07		0.81 ± 0.00		13.54	0 ± 0.02		4.83 ± 0.17			
EGC	EG4	PS99/048-1	MES	78.82	−2.73	1000	−0.24	34.91		13.77 ± 0.04		0.94 ± 0.02		14.65	0 ± 0.01		7.10 ± 0.01			
EGC	EG4	PS99/048-1	BAT	78.82	−2.73	2527	−0.76	34.91		14.97 ± 0.09		1.06 ± 0.01		14.12	0 ± 0.01		12.07 ± 0.19			
N	N3	PS99/054-1	SUR	79.58	5.17	34	3.28	34.40	0.95	8.09 ± 0.06	5.23 ± 0.01	0.72 ± 0.00	0.31 ± 0.00	11.24	1.29 ± 0.00	0.00	3.19 ± 0.05	1.02 ± 0.03		
N	N3	PS99/054-1	EPI	79.58	5.17	100	4.25	35.10		10.11 ± 0.04		0.78 ± 0.00		12.96	0.47 ± 0.01		3.29 ± 0.04			
N	N3	PS99/054-1	MES	79.58	5.17	1000	−0.33	34.10		13.51 ± 0.02		1.05 ± 0.00		12.87	0 ± 0.00		7.25 ± 0.00			
N	N3	PS99/054-1	BAT	79.58	5.17	2500	−0.72	34.92		14.81 ± 0.05		1.09 ± 0.00		13.59	0 ± 0.00		10.74 ± 0.03			
N	N4	PS99/055-1	SUR	79.74	4.51	22	2.66	33.99	2.21	3.33 ± 0.01	6.80 ± 0.01	0.53 ± 0.01	0.49 ± 0.01	6.28	0.29 ± 0.03	0.00	1.65 ± 0.00	2.06 ± 0.01	119	4047
N	N4	PS99/055-1	EPI	79.74	4.51	100	3.94	35.08		10.64 ± 0.00		1.00 ± 0.03		10.64	0.42 ± 0.01		3.90 ± 0.00			
N	N4	PS99/055-7	MES	79.74	4.51	1000	−0.41	34.91		13.96 ± 0.08		0.92 ± 0.00		15.17	0 ± 0.01		9.07 ± 0.19			
N	N4	PS99/055-7	BAT	79.74	4.51	2500	−0.74	34.92		14.47 ± 0.02		0.86 ± 0.00		16.83	0 ± 0.00		11.24 ± 0.14			
N	N5	PS99/053-2	SUR	79.92	3.06	19	0.75	33.59	7.40	0.97 ± 0.01	8.21 ± 0.01	0.51 ± 0.04	0.66 ± 0.02	1.90	0 ± 0.04	1.56 ± 0.01	0.71 ± 0.01	2.34 ± 0.04	14	9401
N	N5	PS99/053-2	EPI	79.92	3.06	100	4.27	35.10		10.05 ± 0.05		1.10 ± 0.06		9.14	0.42 ± 0.01		3.22 0.08			
N	N5	PS99/053-2	MES	79.92	3.06	1000	−0.23	34.91		13.04 ± 0.12		1.34 ± 0.01		9.73	0 ± 0.00		6.50 ± 0.02			
N	N5	PS99/053-2	BAT	79.92	3.06	2427	−0.74	34.92		14.29 ± 0.04		1.59 ± 0.00		8.99	0 ± 0.01		10.79 ± 0.01			
WSC	HG1	PS99/066-2	SUR	79.14	6.09	17	6.27	35.33	3.42											
WSC	HG1	PS99/066-5	EPI	79.14	6.09	100	4.38	35.09												
WSC	HG1	PS99/066-5	MES	79.14	6.09	500	1.46	34.95												
WSC	HG1	PS99/066-5	BAT	79.14	6.09	1253	−0.81	34.91												
WSC	HG2	PS99/057-1	SUR	79.13	4.91	22	2.30	34.90	2.23	6.15 ± 0.01	5.30 ± 0.01	0.89 ± 0.00	0.55 ± 0.02	6.91	0.94 ± 0.00	0.00	2.74 ± 0.02	1.36 ± 0.05		
WSC	HG2	PS99/057-1	EPI	79.13	4.91	100	3.60	35.04		10.84 ± 0.01		1.30 ± 0.01		8.34	0 ± 0.01		3.88 ± 0.11			
WSC	HG2	PS99/057-1	MES	79.13	4.91	1000	−0.58	34.91		14.15 ± 0.09		1.69 ± 0.01		8.37	0 ± 0.01		9.33 ± 0.06			
WSC	HG2	PS99/057-1	BAT	79.13	4.91	1492	−0.81	34.91		14.95 ± 0.02		1.78 ± 0.07		8.40	0 ± 0.00		12.35 ± 0.02			
WSC	HG4	PS99/042-11	SUR	79.07	4.19	28	0.41	34.44	3.54	5.79 ± 0.04	6.42 ± 0.04	0.66 ± 0.02	0.41 ± 0.02	8.77	0.56 ± 0.01	0.17 ± 0.01	2.66 ± 0.02	2.11 ± 0.03	29	4007
WSC	HG4	PS99/042-11	EPI	79.07	4.19	100	3.51	35.04		10.88 ± 0.07		0.87 ± 0.06		12.51	0 ± 0.01		4.16 ± 0.12			
WSC	HG4	PS99/042-1	MES	79.06	4.19	1000	−0.35	34.91		13.71 ± 0.04		0.96 ± 0.01		14.28	0 ± 0.01		7.70 ± 0.02			
WSC	HG4	PS99/042-1	BAT	79.06	4.19	2462	−0.73	34.92		14.67 ± 0.05		1.01 ± 0.00		14.52	0 ± 0.00		11.59 ± 0.03			
WSC	HG5	PS99/044-1	SUR	79.07	3.66	25	1.74	32.363	4.24	5.1 ± 0.03	7.08 ± 0.02	0.53 ± 0.00	0.37 ± 0.00	9.62	0.50 ± 0.02	0.00	3.19 ± 0.01	1.96 ± 0.06		
WSC	HG5	PS99/044-1	EPI	79.07	3.66	100	3.94	35.089		11.26 ± 0.03		0.80 ± 0.00		14.08	0 ± 0.01		4.75 ± 0.03			
WSC	HG5	PS99/044-1	MES	79.07	3.66	2600	−0.73	34.924												
WSC	HG5	PS99/044-1	BAT	79.07	3.66	3038	−0.70	34.925												
WSC	HG7	PS99/046-1	SUR	79.05	3.53	35	3.73	33.957	4.46	5.24 ± 0.01	2.61 ± 0.05	0.51 ± 0.00	0.09 ± 0.00	10.27	0.48 ± 0.00	0.00	2.84 ± 0.20	1.68 ± 0.08		
WSC	HG7	PS99/046-1	EPI	79.05	3.53	100	3.35	35.016		10.35 ± 0.04		0.74 ± 0.00		13.99	0.09 ± 0.00		4.21 ± 0.02			
WSC	HG7	PS99/046-1	MES	79.05	3.53	1005	−0.28	34.908												
WSC	HG7	PS99/046-1	BAT	79.05	3.53	3772	−0.63	34.924												
WSC	HG9	PS99/059-2	SUR	79.13	2.84	24	−1.24	35.089	1.90	1.74 ± 0.04	6.58 ± 0.01	0.67 ± 0.00	0.58 ± 0.13	2.60	0 ± 0.02	0.73 ± 0.21	1.72 ± 0.02	1.96 ± 0.01		
WSC	HG9	PS99/059-2	EPI	79.13	2.84	100	3.92	35.047		10.97 ± 0.01		1.12 ± 0.05		9.79	0.47 ± 0.28		3.70 ± 0.03			
WSC	HG9	PS99/059-2	MES	79.13	2.84	1000	−0.19	34.897		13.59 ± 0.02		1.17 ± 0.17		11.62	0.00 ± 0.17		6.82 ± 0.01			
WSC	HG9	PS99/059-2	BAT	79.13	2.84	2499	−0.72	34.919		15.23 ± 0.01		1.27 ± 0.03		11.99	0.00 ± 0.00		11.11 ± 0.03			

*Chl. a, chlorophyll *a*; EGC, East Greenland current region; N, North region; WSC, West Spitsbergen current region; SUR, surface mixed water; EPI, epipelagic; MES, mesopelagic; BAT, bathypelagic zone; Lat, latitude; Lon, longitude; Temp, temperature; Sal, salinity. Diatoms and *Phaeocystis* spp.*

## Results and Discussion

### Hydrographic and Biogeochemical Conditions Across the Fram Strait

**FIGURE 1:**
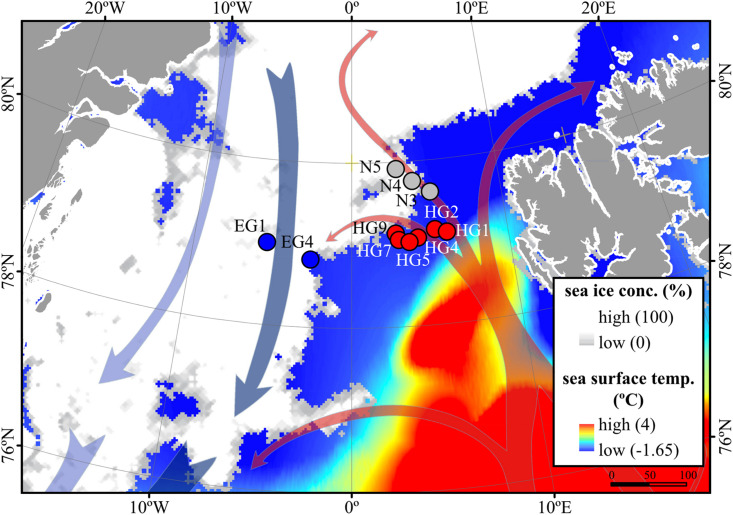
Oceanographic overview of the Fram Strait, including the monthly mean of sea-ice cover and sea surface temperature during July 2016. The sea ice concentration is represented by inverted grayscale (gray = low, white = high). Arrows represent general directions of the West Spitsbergen Current (WSC) (in red) and the East Greenland Current (EGC) (in blue). Stations of water column sampling are indicated and colored according to their sea-ice conditions: ice-covered EGC (EG) stations–blue, ice-margin N stations–gray, ice-free WSC (HG) stations–red. The map was modified from (Fadeev et al., 2020).

Based on the known hydrography of the Strait (Rudels et al., 2012) and the observed sea-ice conditions, we sampled three distinct regions of the Fram Strait (Figure 1): the ice-free eastern part of the Strait (“HG” stations) associated with the WSC (Beszczynska-Möller et al., 2012), the ice-covered western part of the Strait (“EG” stations) associated with the EGC (de Steur et al., 2009), and the partially ice-covered north-eastern part of the Strait (“N” stations) that represents a highly productive ice-margin zone (Hebbeln and Wefer, 1991; Perrette et al., 2011).

At the time of sampling in June–July 2016, the low level of inorganic nutrients above the seasonal pycnocline, and the chlorophyll *a* concentrations, suggested a late stage of the phytoplankton bloom across the Strait (Table 1). Microscopic analyses of phyto- and protozooplankton communities previously conducted in representative stations of each region (LTER HAUSGARTEN stations EG1, EG4, N5, N4, HG4, and S3) at the chlorophyll *a* maximum 10–28 m depth), revealed that the communities at the time of sampling in the ice-covered EG and the ice-margin N stations had a higher abundance of diatoms, in contrast to the ice-free HG stations that had a higher abundance of *Phaeocystis* spp., (Fadeev et al., 2020). These locally defined conditions correspond to an interannual trend of distinct phytoplankton bloom conditions observed in the western ice-covered EGC and the eastern ice-free WSC (Nöthig et al., 2015; Fadeev et al., 2018).

### Surface Water Bacterioplankton Communities Are Affected by Distinct Phytoplankton Bloom Conditions

**FIGURE 2:**
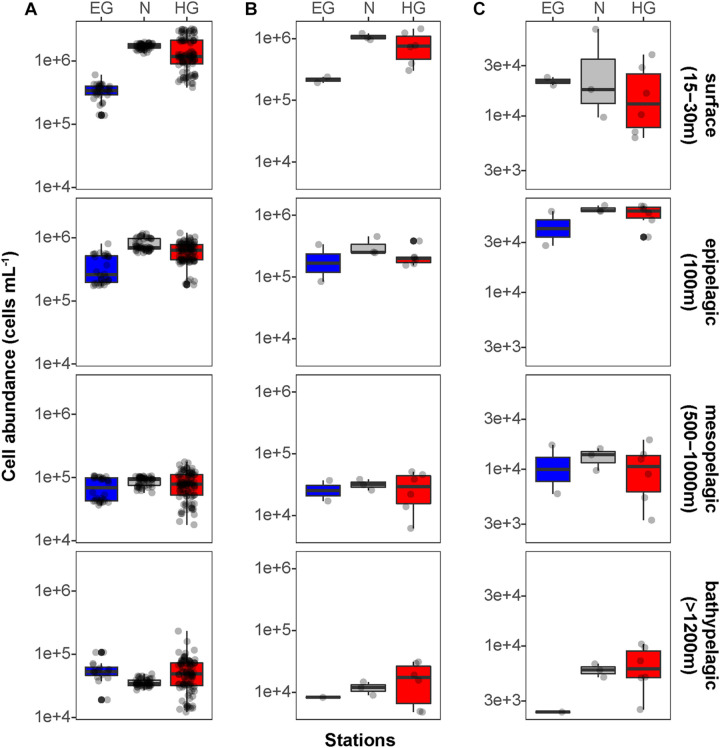
Mean bacterioplankton cell abundances calculated in the different regions of the Fram Strait: Total bacterioplankton in panel **(A)**; *Bacteria* in panel **(B)**; and *Archaea* in panel **(C)**. Box plots were calculated based on cell abundance. Note the different scale of the cell abundances for *Archaea*. The different regions are indicated by color: ice-covered EGC–blue (EG stations), ice-margin N–gray (N stations), ice-free WSC–red (HG stations).

**FIGURE 3:**
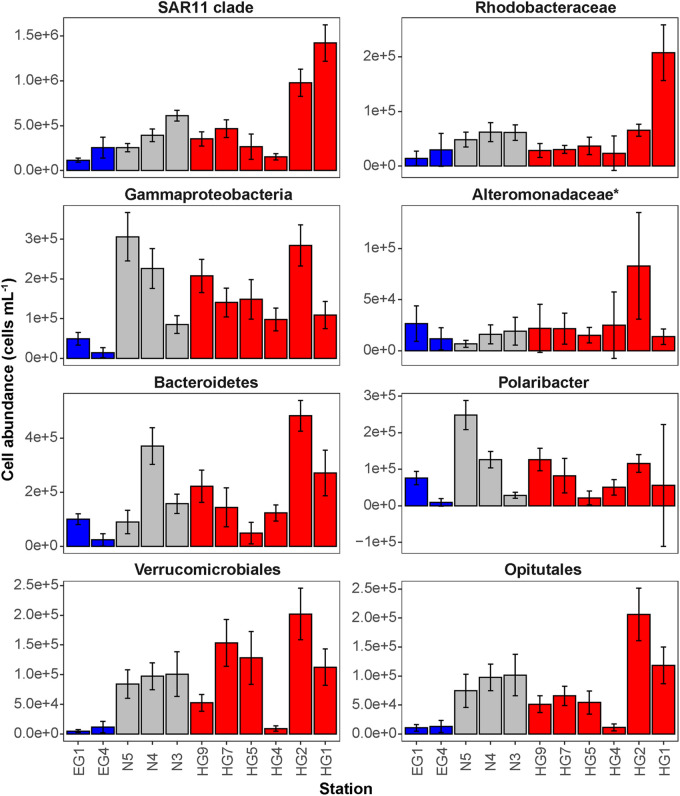
Mean cell abundances of selected taxonomic groups at each station in surface (15–30 m) waters (cells mL^– 1^). The different regions are indicated by color: ice-covered EGC–blue, ice-margin N–gray, ice-free WSC–red.

**FIGURE 4:**
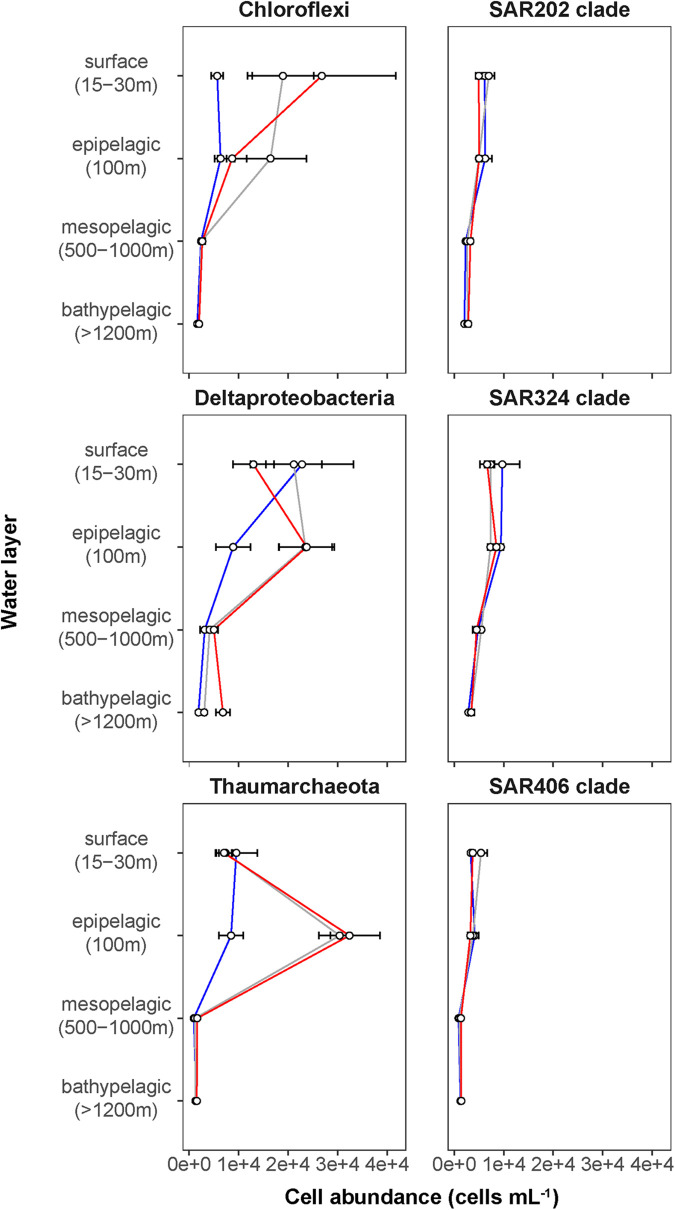
Depth profiles of mean cell abundances of selected taxonomic groups (cells mL^–1^) calculated for the different Fram Strait regions. The different regions are indicated by color: ice-covered EGC–blue (EG stations), ice-margin N–gray (N stations), ice-free WSC–red (HG stations).

Phytoplankton blooms in surface waters generally lead to an increased cell abundance of heterotrophic bacteria that are specialized in the degradation of organic matter from algal exudates and phytodetritus (Buchan et al., 2014; Teeling et al., 2016). Previous observations in Fram Strait revealed a strong influence of the summer phytoplankton bloom conditions on the composition and structure of bacterioplankton communities (Wilson et al., 2017; Müller et al., 2018), differing also between the ice-covered and ice-free regions of the Strait (Fadeev et al., 2018). We observed significantly higher total cell abundances of the bacterioplankton (i.e., all DAPI-stained bacterial and archaeal cells) in the surface water of the HG and N stations (6–17 × 10^5^ cells mL^–1^; Supplementary Table 2), as compared to the EG stations (3 × 10^5^ cells mL^–1^; Kruskal–Wallis test; *χ*^2^ = 81.85, d*f* = 2, *p*-value < 0.01). The communities were dominated by bacterial cells that comprised 8–11 × 10^5^ cells mL^–1^ in the HG and N stations, and 2 × 10^5^ cells mL^–1^ in the EG stations (Figure 2 and Supplementary Table 2). The bacterial communities exhibited high abundance of the classes *Bacteroidetes* (2.1 × 10^5^ cells mL^–1^) in the HG and N stations, followed by *Gammaproteobacteria* (from 1.6 to 2.1 × 10^5^ cells mL^–1^) and *Verrucomicrobia* (from 1.6 to 2.1 × 10^5^ cells mL^–1^), with a several-fold higher cell abundance, compared to the EG stations (where together they comprised between 0.1 and 0.3 × 10^5^ cells mL^–1^) (Figure 3 and Supplementary Table 3). These taxonomic groups were previously suggested to be associated with the seasonal phytoplankton blooms in the region (Wilson et al., 2017; Fadeev et al., 2018). Previous molecular studies also have shown that various taxonomic groups had higher sequence proportion in surface waters of ice-covered, compared to ice-free, regions of the Fram Strait, and are likely associated with Arctic water masses and winter communities in the Fram Strait (Wilson et al., 2017; Fadeev et al., 2018, 2020; Müller et al., 2018). Our microscopy data showed that while *Thaumarchaeota* and the SAR202 clade had only little variations between the different regions, the class *Deltaproteobacteria* and the SAR324 clade exhibited much higher cell abundances in the ice-covered EG stations, as compared to the ice-free HG and ice-margin N stations (Figure 4 and Supplementary Table 3). Hence, the observed patterns in surface water bacterioplankton communities seem to be driven by differences in environmental conditions across the Fram Strait.

In our study, the relative abundance of *Bacteroidetes, Gammaproteobacteria*, and *Verrucomicrobia* were consistent with in 16S rRNA gene observations of size-fractionated bacterioplankton communities (i.e., free-living and particle-associated) conducted during the same expedition (>75%) (Fadeev et al., 2020). However, other taxonomic groups (e.g., *Alteromonadaceae*) showed two- to threefold lower relative abundance in the molecular study (Supplementary Figure 1). These discrepancies can be explained by previously conducted direct methodological comparison between 16S rRNA gene observations and CARD-FISH counts (Fadeev et al., 2021), which suggested potential over-representation of the SAR11 clade in the microscopy counts that could affect the proportional representation of other taxonomic groups in the dataset. Alternatively, the potentially higher cellular activity (and thus higher ribosomal content) of phytoplankton bloom-associated taxonomic groups (e.g., *Bacteroidetes*) may have altered their representation in the PCR-based 16S rRNA gene dataset (Rosselli et al., 2016), and thus potentially lower sequence proportion of other taxonomic groups. The methodology applied in this study avoids this compositionality effect and allows for the direct determination of absolute cell abundances of each targeted taxonomic group.

To test the hypothesis that environmental conditions across the Strait shape bacterioplankton communities, we examined a set of key physicochemical environmental parameters that represent the distinct water masses (temperature and salinity) and the different phytoplankton bloom conditions (categorized based on chlorophyll *a* concentration and consumed inorganic nutrients) across the Strait. We did not find significant correlations between these physical and biogeochemical parameters, which suggest to some extent their independent effect on the bacterioplankton communities (Supplementary Figure 2). Based on this assumption we conducted specific correlation tests between each of these environmental parameters and cell abundances of various taxonomic groups (Supplementary Table 4). Cell abundances of *Verrucomicrobia* and its order *Opitutales*, as well as of the SAR11 clade and the family *Rhodobacteraceae* (both members of the class *Alphaproteobacteria*), showed significant positive correlations to water temperature (Pearson’s correlation; *r* > 0.5, *p*-value < 0.05; Supplementary Table 4), suggesting an association with the warmer Atlantic waters of the eastern Fram Strait. The *Verrucomicrobia* has been previously shown to be a major polysaccharide-degrading bacterial taxonomic group in the north-western Svalbard fjord Smeerenburgfjord (Cardman et al., 2014), and therefore may also be associated with the outflow from the Svalbard fjords (e.g., Kongsfjord) into the Atlantic waters of the WSC (Cottier et al., 2005) sampled for this study. The SAR11 clade and the *Rhodobacteraceae* have both been previously shown to correlate with temperature at high latitudes (Giebel et al., 2011; Tada et al., 2013), and are known to have distinct phylotypes in water masses with different temperatures (Selje et al., 2004; Sperling et al., 2012; Giovannoni, 2017). However, the *Rhodobacteraceae* are also known for their broad abilities in utilizing organic compounds (Buchan et al., 2014; Luo and Moran, 2014). Thus, one cannot rule out that their higher cell abundances in warmer waters of the HG and N stations are associated with the late stage of the phytoplankton bloom and their exudates. In addition, the SAR324 clade (*Deltaproteobacteria*) showed strong positive correlation with statistical significance to salinity (Pearson’s correlation; *r* > 0.5, *p*-value < 0.05; Supplementary Table 4). During the summer, with increased melting of sea ice, a low-salinity water layer is formed in surface waters, and the strong stratification of this water layer enhances the development of the phytoplankton bloom (Fadeev et al., 2018). Consequently, the correlation of SAR324 with higher salinity suggests that their cell abundances are lower in surface waters where, in turn, we observe a strong phytoplankton bloom (e.g., in WSC).

The distinct surface water masses in the region differ not only in their physical but also in their biogeochemical characteristics (Wilson and Wallace, 1990; Fadeev et al., 2018), with higher concentrations of inorganic nitrogen and phosphate in the Atlantic, compared to the Arctic water masses. At the time of sampling, the typical Redfield ratio between inorganic nitrogen (mainly nitrate NO_3_) and inorganic phosphate (PO_4_) was below 16 (Redfield, 1963; Goldman et al., 1979). This suggests that surface waters across all three regions were nitrogen limited (Table 1) due to the progressing phytoplankton growth (Nöthig et al., 2015). In order to disentangle the effect of biological consumption of nutrients from water mass-specific nutrient signatures, we calculated the seasonal net consumption of inorganic nutrients, as the proxy for phytoplankton bloom conditions (Table 1). Consumed nitrate (ΔNO_3_) and phosphate (ΔPO_4_) revealed a very strong positive correlation with statistical significance (Pearson’s correlation; *r* = 0.86, *p*-value < 0.05; Supplementary Table 4). The consumed silica (ΔSiO_3_), used by diatoms, did not show a significant correlation to ΔPO_4_ and ΔNO_3_. This further supports the impact of different phytoplankton populations across the Strait (i.e., diatoms vs. *Phaeocystis*; Fadeev et al., 2020). Phytoplankton bloom-associated environmental parameters (chlorophyll *a* concentration and the consumed inorganic nutrients) revealed weaker relationships with cell abundances of different taxonomic groups (Supplementary Table 4). Furthermore, we did not observe significant positive correlations of the cell abundances of diatoms or *Phaeocystis* spp., with the quantified bacterioplankton taxa. This might be explained by time lags and local differences in the dynamic development of phytoplankton blooms across the entire Strait (Wilson et al., 2017; Fadeev et al., 2018).

### Bacterioplankton Communities Strongly Change in Cell Abundance and Composition With Depth

The complexity of Fram Strait surface waters with different ice-coverages, a dynamic ice-melt water layer and mesoscale mixing events of Atlantic and Polar water masses by eddies (Wekerle et al., 2017), challenges the identification of specific associations between microbial cell abundances and environmental parameters. Some taxonomic groups (e.g., SAR11 clade) were potentially more influenced by the physical processes such as the presence of ice and distinct Arctic water masses (Kraemer et al., 2020). Likely the mixture of all these environmental variables shaped the observed bacterioplankton communities. We found that cell abundances of some taxonomic groups (e.g., *Gammaproteobacteria*) were higher in some stations with more advanced phytoplankton bloom conditions. However, as we have only limited observations of phytoplankton for this study, we cannot test previous hypotheses of direct associations between the abundances of specific phytoplankton groups and bacterioplankton taxa (Fadeev et al., 2018). Nonetheless, the here observed patterns could represent an enhanced growth of the bacterioplankton on algal exudates (Tada et al., 2011; Teeling et al., 2012). Alternatively, considering the advection of Atlantic waters (Wekerle et al., 2017), it is also possible that some of the observed trends represent lateral transport of phytoplankton or bacterioplankton, or both, from the southern part of the Strait.

In surface waters of all stations, ca. 60% of the total bacterioplankton community was covered by the *Bacteria*-specific probes (EUB388 I–III) and up to 8% was covered by the *Archaea*-specific probe (ARCH915; Supplementary Table 2). At depth (>100 m), the coverage of total cells by the *Bacteria*-specific probes strongly decreased to 16–40% of DAPI-stained cells (ANOVA; *F*_3_ = 15.39, *p* < 0.01), while the coverage by the *Archaea*-specific probe significantly increased up to 17% of DAPI-stained cells (ANOVA; *F*_3_ = 34.31, *p* < 0.01; Supplementary Table 2). A similar decrease in detectability of the *Bacteria* -specific probes was previously observed in other bacterioplankton microscopy studies (Karner et al., 2001; Herndl et al., 2005; Varela et al., 2008), and reasons may lie in a ribosomal nucleic acid concentration decrease within the bacterial cells (i.e., lower activity) toward the oligotrophic depths. In addition, there is a potential increase with greater water depths of microbial phylogenetic groups that are not captured by the currently existing probes (Hewson et al., 2006; Galand et al., 2009a; Agogué et al., 2011; Welch and Huse, 2011; Salazar et al., 2016).

We found that in all three regions, total cell abundances of the entire bacterioplankton community were highest at surface with 10^5^–10^6^ cells mL^–1^, and significantly decreased with depth down to 10^4^ cells mL^–1^ at meso- and bathypelagic depths (Figure 2A and Supplementary Table 3; Kruskal–Wallis test; *χ*^2^ = 554.39, d*f* = 3, *p*-value < 0.01). Members of the domain *Bacteria* dominated the communities throughout the entire water column, with highest cell abundances in surface waters (10^5^–10^6^ cells mL^–1^), and significantly lower 10^4^ cells mL^–1^ at depth (Figure 2B; Kruskal–Wallis test; *χ*^2^ = 35.27, d*f* = 3, *p*-value < 0.01). Archaeal cells had an overall lower abundance than bacterial cells by an order of magnitude throughout the entire water column, ranging from 10^4^ cells mL^–1^ at surface down to 10^3^ cells mL^–1^ in bathypelagic waters (Figure 2C). However, unlike *Bacteria*, archaeal communities doubled their absolute cell abundances from ca. 3 × 10^4^ cells mL^–1^ at surface to ca. 6 × 10^4^ cells mL^–1^ at 100 m depth, followed by a significant decrease in cell abundance at meso- and bathypelagic depths (Kruskal–Wallis test; *χ*^2^ = 29.04, d*f* = 3, *p*-value < 0.01). Compared to the stronger decline in bacterial cell numbers, this pattern mirrors the known global trend of relative archaeal enrichment in epipelagic waters (Karner et al., 2001; Herndl et al., 2005; Kirchman et al., 2007; Varela et al., 2008; Schattenhofer et al., 2009), and was also observed in other regions of the Arctic Ocean (Amano-Sato et al., 2013). Altogether, the here observed bacterioplankton cell abundances in surface waters were well within the range of previous observations in the Fram Strait waters, conducted by flow cytometry (Piontek et al., 2014; Fadeev et al., 2018; Engel et al., 2019). However, compared to recent CARD-FISH based observations in eastern Fram Strait (Quero et al., 2020), our cell abundances were consistently one order of magnitude lower along the entire water column. The discrepancy might be associated with methodological differences, such as shorter staining times and the usage of an automated over a manual counting approach in our study. Nevertheless, both studies showed a similar pattern of a strong decrease in bacterioplankton cell abundances with depth, which also matches observations in other oceanic regions (Karner et al., 2001; Church et al., 2003; Teira et al., 2004; Schattenhofer et al., 2009; Dobal-Amador et al., 2016).

### Enigmatic Microbial Lineages Increase in Cell Abundance Toward the Deep Ocean

The deep waters of the Fram Strait basin (>500 m) have a rather homogeneous hydrography (von Appen et al., 2015), and are less affected by the seasonal dynamics that govern the surface layers (Wilson et al., 2017). Previous molecular observations of the deep water bacterioplankton communities showed high sequence abundances of largely unknown taxonomic groups, such as the SAR202 (class *Dehalococcoidia*), SAR324 (class *Deltaproteobacteria*), and SAR406 (phylum *Marinimicrobia*) (Wilson et al., 2017; Fadeev et al., 2020; Quero et al., 2020). There was also higher archaeal sequence abundance at depth, with the class *Nitrososphaeria* (i.e., *Thaumarchaeota*) reaching up to 15% of the sequences in mesopelagic waters (>200 m) (Wilson et al., 2017; Müller et al., 2018; Fadeev et al., 2020). However, it has also been recently shown that in ice-covered regions of the Strait surface-dominant taxonomic groups, such as *Gammaproteobacteria* and *Nitrososphaeria*, are exported via fast-sinking aggregates from surface to the deep ocean (>1,000 m), where they may realize an ecological niche (Fadeev et al., 2020). We observed that in all meso- and bathypelagic waters across all analyzed regions the total cell abundances of the bacterioplankton communities were in the range of 10^4^ cells mL^–1^ (Figure 2), reflecting observations made in Arctic mesopelagic waters (Wells et al., 2006; Quero et al., 2020). Bacterial taxonomic groups that dominated the surface water communities (e.g., *Bacteroidetes*, *Gammaproteobacteria*, and *Verrucomicrobia*), in both ice-free and ice-covered regions of the Strait, decreased by two orders of magnitude in their cell abundances at meso- and bathypelagic depths (Kruskal–Wallis test; *p*-value < 0.01; Figure 3 and Supplementary Table 3). This trend strongly correlated with the total bacterioplankton cell abundances along the general water column (Pearson’s correlation; *r* > 0.8, *p*-value < 0.05 and Supplementary Table 4). In contrast, other bacterial groups, such as the SAR202 and SAR324 clades, proportionally increased in cell abundances with depth and maintained overall constant cell abundances of ca. 0.5 × 10^4^ cells mL^–1^ until the deep basin (Supplementary Table 3). Previous molecular studies of bacterioplankton communities in the Fram Strait suggested a proportional increase of these largely understudied bacterial lineages in the deep ocean, which were previously found to be associated with winter (surface) bacterioplankton (Wilson et al., 2017; Fadeev et al., 2020). The cell abundances presented here indicate that their increasing proportional abundance at depth is due to stronger decrease in the cell abundances of other groups (Figure 4 and Supplementary Table 3). Very little is currently known about these two taxonomic groups, but previous genetic observations suggest that they possess distinct metabolic capabilities, and may be involved in the degradation of recalcitrant organic matter (SAR202 clade; Landry et al., 2017; Colatriano et al., 2018; Saw et al., 2019), or, in sulfur oxidation (SAR324 clade; Swan et al., 2011; Sheik et al., 2014). Their homogeneous distribution from the stratified surface to the homogenous deep ocean suggests that through high functional plasticity these enigmatic bacterial groups fulfill various ecological niches throughout the water column (Saw et al., 2019; Wei et al., 2020), and thus may play important roles in oceanic nutrient cycling.

With depth, the decrease of archaeal cell abundances was less than that of members of the domain *Bacteria* (Figure 4 and Supplementary Table 3), meaning that members of the *Archaea* were proportionally increasing in the total microbial deep-water communities. The *Thaumarchaeota* strongly correlated with the pattern of the archaeal cell abundances (Pearson’s correlation; *r* = 0.76, *p*-value < 0.05; Supplementary Table 4), showing a two-fold increase in cell abundance from surface to epipelagic depth (100 m), followed by a substantial decrease toward meso- and bathypelagic waters (Figure 4 and Supplementary Table 3). This two-fold increase toward the epipelagic depths corresponds to previous observations of *Thaumarchaeota* in the north Atlantic (Müller et al., 2018), and a further increase in their cell abundances at higher depths (>1,000 m) was also observed in other oceanic regions (Karner et al., 2001; Church et al., 2003; Herndl et al., 2005; Teira et al., 2006; Galand et al., 2009b). It has been shown in molecular studies that *Thaumarchaeota* comprise a large proportion of the bacterioplankton communities in the Fram Strait, especially in the epipelagic waters (Wilson et al., 2017; Müller et al., 2018; Fadeev et al., 2020). In our study, the *Thaumarchaeota* exhibited their highest cell abundances at 100 m in the ice-free HG, and at the ice-margin N stations (3 × 10^4^ cells mL^–1^), where they comprised half of the total archaeal community (Figure 4 and Supplementary Table 3). The strong absolute decrease of *Thaumarchaeota* cell abundances toward the meso- and bathypelagic waters suggests a decrease in cell number or activity with depth (Herndl et al., 2005; Kirchman et al., 2007; Alonso-Sáez et al., 2012), and thus lower cell detectability. In deeper water layers, other pelagic archaeal groups, such as the phylum *Euryarchaeota* that was not quantified in this study, may increase in abundance and form the bulk of total archaeal cells here (Galand et al., 2010; Fadeev et al., 2020).

## Conclusion

Using state-of-the-art semi-automatic microscopy cell counting, we quantified the absolute cell abundance of 12 key taxonomic groups in summer bacterioplankton communities of both ice-free and ice-covered regions of the Fram Strait. We found that in surface waters some taxonomic groups were associated with the distinct water masses of the Strait (e.g., *Rhodobacteraceae* with the Atlantic waters). Surface water bacterioplankton communities were dominated by *Gammaproteobacteria*, *Bacteroidetes*, and *Verrucomicrobia*, which corresponded with biogeochemical conditions in the ongoing seasonal phytoplankton bloom. This suggests that currently predicted longer seasonal phytoplankton blooms, as well as the increasing Atlantic influence on the Arctic Ocean (i.e., “Atlantification”), may have a strong impact on the composition and biogeographical distribution of certain bacterioplankton taxonomic groups in the surface Arctic waters.

This study also provides the first extensive quantification of bacterioplankton community standing stocks in the deep Arctic water column (>500 m). With depth, some taxonomic groups, such as the SAR202 clade, maintained similar abundances throughout the entire water column (2,500 m depth), where other taxa decline by several-fold. The observation of a homogenous abundance further supports the previously established hypothesis that through high functional plasticity these taxonomic groups are realizing various ecological niches throughout the entire water column.

Altogether, our quantitative data on cell abundances of ecologically relevant taxonomic bacterioplankton groups provide insights into factors structuring pelagic bacterioplankton communities from surface to the deep waters of the Arctic Ocean, and add to a baseline to better assess future changes in a rapidly warming region.

## Materials and Methods

### Sampling and Environmental Data Collection

Sampling was carried out during the RV Polarstern expedition PS99.2 to the Long-Term Ecological Research (LTER) site HAUSGARTEN in Fram Strait (June 24th–July 16th, 2016). Sampling was carried out with 12 L Niskin bottles mounted on a CTD rosette (Sea-Bird Electronics Inc. SBE 911 plus probe) equipped with temperature and conductivity sensors, a pressure sensor, altimeter, and a chlorophyll fluorometer. In ice-covered regions the samples were collected through holes in the ice kept open by the research vessel. On board, the samples were fixed with formalin in a final concentration of 2% for 10–12 h, then filtered onto 0.2 μm polycarbonate Nucleopore Track-Etched filters (Whatman, Buckinghamshire, United Kingdom), and stored at −20°C for further analysis.

Hydrographic data of the seawater including temperature and salinity were retrieved from PANGEA (Schröder and Wisotzki, 2014), along with measured chlorophyll *a* concentration (Nöthig et al., 2018; Fadeev et al., 2020) (Table 1).

Relative abundance of relevant 16S rRNA as well as data on microscopic abundances of microbial eukaryotes in phytoplankton blooms of the sample location was obtained from (Fadeev et al., 2020).

### Catalyzed Reporter Deposition-Fluorescence *in situ* Hybridization (CARD-FISH)

We quantified absolute cell abundances of 12 key bacterioplankton groups (Supplementary Table 1), members of the *Bacteria* and *Archaea*, based on their relatively high sequence abundance and recurrences in previous molecular studies of Arctic waters (Bowman et al., 2012; Wilson et al., 2017; Müller et al., 2018; Fadeev et al., 2020). The selected probes covered a variety of taxonomic entities to address standing stocks at different taxonomic levels. All probes were checked for specificity and coverage of their target groups against the SILVA database release 132 (Quast et al., 2013). CARD-FISH was applied based on the protocol established by (Pernthaler et al., 2002), using horseradish-peroxidase (HRP)–labeled oligonucleotide probes (Biomers.net, Ulm, Germany). All filters were embedded in 0.2% low-gelling-point agarose, and treated with 10 mg mL^–1^ lysozyme solution (Sigma-Aldrich Chemie GmbH, Hamburg, Germany) for 1 h at 37°C. Filters for enumerating *Archaea* and *Thaumarchaeota* were treated for an additional 30 min in 36 U mL^–1^ achromopeptidase (Sigma-Aldrich Chemie GmbH, Hamburg, Germany) and 15 μg mL^–1^ proteinase K at 37°C. Subsequently, endogenous peroxidases were inactivated by submerging the filter pieces in 0.15% H_2_O_2_ in methanol for 30 min before rinsing in Milli-Q water and dehydration in 96% ethanol. Then, the filters were covered in hybridization buffer and a probe concentration of 0.2 ng μL^–1^. Hybridization was performed at 46°C for 2.5 h, followed by washing in pre-warmed washing buffer at 48°C for 10 min, and 15 min in 1x PBS. Signal amplification was carried out for 45 min at 46°C with amplification buffer containing either tyramide-bound Alexa 488 (1 μg/mL) or Alexa 594 (0.33 μg mL^–1^). Afterward, the cells were counterstained in 1 μg/mL DAPI (4′,6-diamidino-2-phenylindole; Thermo Fisher Scientific GmbH, Bremen, Germany) for 10 min at 46°C. After rinsing with Milli-Q water and 96% ethanol, the filter pieces were embedded in a 4:1 mix of Citifluor (Citifluor Ltd., London, United Kingdom) and Vectashield (Vector Laboratories, Inc., Burlingame, CA, United States), and stored overnight at −20°C for later microscopy evaluation.

### Automated Image Acquisition and Cell Counting

The filters were evaluated microscopically under a Zeiss Axio Imager.Z2 stand (Carl Zeiss MicroImaging GmbH, Jena, Germany), equipped with a multipurpose fully automated microscope imaging system (MPISYS), a Colibri LED light source illumination system, and a multi-filter set 62HE (Carl Zeiss MicroImaging GmbH, Jena, Germany). Pictures were taken via a cooled charged-coupled-device (CCD) camera (AxioCam MRm; Carl Zeiss AG, Oberkochen, Germany) with a 63 × oil objective, a numerical aperture of 1.4, and a pixel size of 0.1016 μm/pixel, coupled to the AxioVision SE64 Rel.4.9.1 software (Carl Zeiss AG, Oberkochen, Germany) as described by Bennke et al. (2016). Exposure times were adjusted after manual inspection with the AxioVision Rel.4.8 software coupled to the SamLoc 1.7 software (Zeder et al., 2011), which was also used to define the coordinates of the filters on the slides. For image acquisition, channels were defined with the MPISYS software, and a minimum of 55 fields of view with a minimum distance of 0.25 mm were acquired of each filter piece by recoding a z-stack of seven images in autofocus.

Cell enumeration was performed with the software Automated Cell Measuring and Enumeration Tool (ACMETool3, 2018-11-09; M. Zeder, Technobiology GmbH, Buchrain, Switzerland). Total bacterioplankton cells were determined as the total amount of DAPI-stained cells. Counts for each taxonomic group included only cells that were simultaneously stained by DAPI and the taxa-specific FISH probe.

### Calculation of Consumed Inorganic Nutrients

Following (Fadeev et al., 2018) the nutrient consumption (Δ) at each station was calculated by subtracting the mean value of all collected measurements above 50 m from the mean value of all collected measurements between 50 and 100 m (below the seasonal pycnocline).

### Statistical Analyses

All statistical analyses and calculations in this study were performed using R (v4.0.2) (www.r-project.org) in RStudio (v1.3.1056), *i.e.*, statistical tests for normality, ANOVA and Kruskal–Wallis. *Post hoc* Wilcoxon test and Pearson’s rank correlation coefficient were conducted with the R package “rstatix” (v0.6.0) (Kassambara, 2020). Plots were generated using the R package “ggplot2” (v3.3.2) (Wickham, 2016) and “tidyverse” (v1.3.0) (Wickham et al., 2019).

## Data Availability Statement

The raw data supporting the conclusions of this article will be made available by the authors, without undue reservation.

## Author Contributions

MC-M, EF, and VS-C designed and conducted the study, and wrote the manuscript with guidance from AB. MC-M performed the hybridizations, cell counting, data and statistical analysis with guidance from VS-C (probe selection, CARD-FISH application, and counting) and EF (data and statistical analysis). All authors critically revised the manuscript and gave their approval of the submitted version.

## Conflict of Interest

The authors declare that the research was conducted in the absence of any commercial or financial relationships that could be construed as a potential conflict of interest.
