# Supplementary material for: Spatial Distribution of Arctic Bacterioplankton Abundance Is Linked to Distinct Water Masses and Summertime Phytoplankton Bloom Dynamics (Fram Strait, 79°N)
Source: Front Microbiol. 2021 May 10;12:658803. doi: 10.3389/fmicb.2021.658803 (PMC8143376; doi:10.3389/fmicb.2021.658803)
Supplement: Supplementary file 1 [file Data_Sheet_1.doc]

Supplementary Material

# Spatial distribution of Arctic bacterioplankton abundance is linked to distinct water masses and summertime phytoplankton bloom dynamics (Fram Strait, 79°N)

**Magda G. Cardozo-Mino, Eduard Fadeev, Verena Salman-Carvalho, Antje Boetius**

**Table S1.** Specificities of publicly available rRNA-targeting oligonucleotide probes used during CARD-FISH for the quantification of pelagic microbial groups in the Fram Strait, and their total sequence proportions in 16S rRNA data of Fadeev et al. (2020). FA - formamide concentration in the hybridization buffer.

*Taxonomic groups in the 16S rRNA data were defined according to SILVA reference database v138 (k - kingdom, p - phylum, c - class, o - order, f - family, g - genus).

| **Probe name** | **Targeted group** | **Sequence proportion*** | **Sequence (5’-3’)** | **FA (%)** | **Reference** |
| --- | --- | --- | --- | --- | --- |
| EUB338 I | *Bacteria* | 92% (k: *Bacteria*) | GCT GCC TCC CGT AGG AGT | 35 | (Amann et al., 1990) |
| EUB338 II | *Planctomycetales (Bacteria)* | 1% (o: *Planctomycetales*) | GCA GCC ACC CGT AGG TGT | 35 | (Daims et al., 1999) |
| EUB338 III | *Verrucomicrobia and Bacteria* | 9% (p: *Verrucomicrobiae*) | GCT GCC ACC CGT AGG TGT | 35 | (Daims et al., 1999) |
| Non338 | nonsense probe | - | ACT CCT ACG GGA GGC AGC | 35 | (Wallner et al., 1993) |
| ARCH915 | *Archaea* | 8% (k: *Archaea*) | GTG CTC CCC CGC CAA TTC CT | 35 | (Amann et al., 1995) |
| CFX1223 | *Chloroflexi* | 2% (p: *Chloroflexi*) | CCA TTG TAG CGT GTG TGT MG | 35 | (Björnsson et al., 2002) |
| GNSB941 | *Chloroflexi* |  | AAA CCA CAC GCT CCG CT | 35 | (Gich et al., 2001) |
| SAR202-312R | SAR202 clade | 1% (o: SAR202 clade) | TGT CTC AGT CCC CCT CTG | 40 | (Morris et al., 2004) |
| PSA184 | *Alteromonadaceae, Colwelliaceae, Pseudoalteromonadaceae* | 2% (o: *Alteromonadales*) | CCC CTT TGG TCC GTA GAC | 30 | (Eilers et al., 2000) |
| GAM42a | *Gammaproteobacteria* | 22% (c: *Gammaproteobacteria*) | GCC TTC CCA CAT CGT TT | 35 | (Manz et al., 1992) |
| BET421 | competitor for GAM42a |  | GCC TTC CCA CTT CGT TT | 35 | (Manz et al., 1992) |
| POL740 | *Polaribacter* | 11% (g: *Polaribacter*) | CCC TCA GCG TCA GTA CAT ACG T | 35 | (Malmstrom et al., 2007) |
| CF968 | *Bacteroidetes* | 28% (c: *Bacteroidia*) | GGT AAG GTT CCT CGC GTA | 55 | (Acinas et al., 2015) |
| Opi346 | *Opitutales* | 7% (o: *Opitutales*) | TTC GAA ACT GCT GCC ACC C | 20 | (Reintjes 2017) |
| Cren554 | *Thaumarchaeota* | 6% (c: *Nitrososphaeria*) | TTA GGC CCA ATA ATC MTC CT | 20 | (Massana et al., 1997) |
| SAR406-97 | SAR406 clade  (*Marinimicrobia*) | 4% (p: SAR406 clade) | CAC CCG TTC GCC AGT TTA | 40 | (Fuchs et al., 2005) |
| DELTA495a | *Deltaproteobacteria* | 1% (p: *Bdellovibrionota*, *Desulfobacterota*, *Myxococcota*) | AGT TAG CCG GTG CTT CCT | 35 | (Loy et al., 2002) |
| cDELTA495a | competitor for DELTA495a |  | AGT TAG CCG GTG CTT CTT | 35 | (Lücker et al., 2007) |
| DELTA495b | *Deltaproteobacteria* |  | AGT TAG CCG GCG CTT CCT | 35 | (Loy et al., 2002) |
| cDELTA495b | competitor for DELTA495b |  | AGT TAG CCG GCG CTT CKT | 35 | (Lücker et al., 2007) |
| DELTA495c | *Deltaproteobacteria* |  | AAT TAG CCG GTG CTT CCT | 35 | (Loy et al., 2002) |
| cDELTA495c | competitor for DELTA495c |  | AAT TAG CCG GTG CTT CTT | 35 | (Lücker et al., 2007) |
| SAR324-R-625 | SAR324 clade  (“Marine group B”) | 3% (p: SAR324 clade) | CGA AAG ACC CTC CGG | 15 | (Wright et al., 1997) |
| ROS536 | *Rhodobacteraceae* | 2% (f: *Rhodobacteraceae*) | CAA CGC TAA CCC CCT CCG | 35 | (Brinkmeyer et al., 2000) |
| SAR11-152R | SAR11 clade | 9% (o: SAR11 clade) | TTAGCACAAGTTTCCYCGTGT | 25 | (Morris et al. 2002) |
| SAR11-441R | SAR11 clade |  | TACAGTCATTTTCTTCCCCGAC | 25 | (Morris et al. 2002) |
| SAR11-441Rmod | SAR11 clade |  | TACCGTCATTTTCTTCCCCGAC | 25 | (Gomez-Pereira et al., 2013) |
| SAR11-542R | SAR11 clade |  | TCCGAACTACGCTAGGTC | 25 | (Morris et al. 2002) |
| SAR11-732R | SAR11 clade |  | GTCAGTAATGATCCAGAAAGYTG | 25 | (Morris et al. 2002) |
| SAR11-487Rmodif | SAR11 clade |  | CGGACCTTCTTATTCGGG | 25 | (Gomez-Pereira et al., 2013) |
| SAR11-487_h3 | SAR11 clade |  | CGGCTGCTGGCACGAAGTTAGC | 25 | (Gomez-Pereira et al., 2013) |

**Table S2**. Average bacterioplankton cell abundances along the water column of ice-covered and ice-free regions of the Fram Strait. The DAPI counts represent total bacterioplankton cell abundances. The proportions (%) of *Archaea* (ARCH) and *Bacteria* (EUB) were calculated based on the total bacterioplankton cell abundances (DAPI stained cells). Sample size ‘n’ represents the number of counted fields of view. Standard error was not calculated for samples of the EGC located in the bathypelagic zone due to one station located at this depth in the region (NA). EGC: the ice-covered East Greenland Current, EG stations, N: the marginal ice northern stations, WSC: the ice-free West Spitsbergen Current, HG stations. All values are represented in 10^5^ cells mL^-1^.

| **Region** | **Water layer** | **DAPI** | ***Archaea*** | **%** | ***n*** | ***Bacteria*** | **%** | ***n*** |
| --- | --- | --- | --- | --- | --- | --- | --- | --- |
| EGC | Surface | 3.4 ± 0.2 | 0.2 ± 0.0 | 8 | 56 | 2.2 ± 0.2 | 60 | 52 |
| EGC | Epipelagic | 3.5 ± 1.2 | 0.4 ± 0.2 | 14 | 57 | 2.1 ± 1.3 | 55 | 48 |
| EGC | Mesopelagic | 0.7 ± 0.3 | 0.1 ± 0.1 | 17 | 51 | 0.2 ± 0.1 | 40 | 44 |
| EGC | Bathypelagic | 0.6 ± NA | 0.02 ± NA | 12 | 32 | 0.1 ± NA | 16 | 32 |
| N | Surface | 17.1 ± 0.7 | 0.3 ± 0.1 | 2 | 90 | 10.7 ± 0.7 | 62 | 81 |
| N | Epipelagic | 7.9 ± 1.2 | 0.6 ± 0.0 | 9 | 90 | 3.2 ± 0.7 | 40 | 73 |
| N | Mesopelagic | 0.9 ± 0.1 | 0.1 ± 0.0 | 17 | 77 | 0.3 ± 0.0 | 37 | 101 |
| N | Bathypelagic | 0.4 ± 0.0 | 0.1 ± 0.0 | 17 | 63 | 0.1 ± 0.0 | 37 | 85 |
| WSC | Surface | 15.0 ± 3.6 | 0.2 ± 0.0 | 1 | 146 | 8.1 ± 1.8 | 59 | 150 |
| WSC | Epipelagic | 6.2 ± 0.7 | 0.6 ± 0.1 | 12 | 217 | 2.2 ± 0.3 | 36 | 175 |
| WSC | Mesopelagic | 0.8 ± 0.1 | 0.1 ± 0.0 | 13 | 152 | 0.3 ± 0.1 | 34 | 166 |
| WSC | Bathypelagic | 0.5 ± 0.1 | 0.1 ± 0.0 | 15 | 198 | 0.2 ± 0.0 | 33 | 201 |

**Table S3. Cell abundances and proportions of taxonomic groups in the different regions and water layers across the Fram Strait. The proportions (%) were calculated based on the total bacterioplankton cell abundances (DAPI stained cells), ‘n’ represents the number of counted fields of view. All values are represented in 10^5^ cells mL^-1^. *Alteromonadaceae/Colwelliaceae/Pseudoalteromonadaceae* (ATL), *Bacteroidetes* (BACT), *Chloroflexi* (CFX)*, Thaumarchaeota* (THA), *Deltaproteobacteria* (DELTA)*, Gammaproteobacteria* (GAM), *Opitutales* (OPI)*, Polaribacter* (POL)*, Rhodobacteraceae* (ROS), *Verrucomicrobia* (VER), SAR202, SAR324, SAR406 and SAR11 clades.**

| **Region** | **Station** | **Water layer** | **ALT** | **%** | ***n*** | BACT | **%** | ***n*** | CFX | **%** | ***n*** | **THA** | **%** | ***n*** | DELTA | **%** | ***n*** | GAM | **%** | ***n*** | OPI | **%** | ***n*** |
| --- | --- | --- | --- | --- | --- | --- | --- | --- | --- | --- | --- | --- | --- | --- | --- | --- | --- | --- | --- | --- | --- | --- | --- |
| EGC | EG1 | SRF | 0.27 ± 0.17 | 6.2 | 35 | 1.01 ± 0.20 | 23.7 | 31 | 0.07 ± 0.06 | 2.4 | 25 | 0.05 ± 0.03 | 1.6 | 15 | 0.33 ± 0.20 | 8.9 | 42 | 0.49 ± 0.16 | 14.6 | 35 | 0.11 ± 0.06 | 2.7 | 32 |
| EGC | EG1 | EPI | 0.06 ± 0.04 | 3.2 | 28 | 0.13 ± 0.07 | 7.5 | 27 | 0.05 ± 0.02 | 2.3 | 20 | 0.06 ± 0.03 | 3.2 | 32 | 0.05 ± 0.02 | 2.7 | 33 | 0.11 ± 0.07 | 6.0 | 36 | 0.16 ± 0.12 | 1.9 | 26 |
| EGC | EG1 | MESO | 0.02 ± 0.01 | 4.2 | 20 | 0.01 ± 0.00 | 2.1 | 4 | 0.03 ± 0.01 | 6.8 | 28 | 0.01 ± 0.00 | 2.1 | 8 | 0.02 ± 0.01 | 5.1 | 27 | 0.01 ± 0.00 | 2.2 | 5 | 0.02 ± 0.01 | 3.7 | 16 |
| EGC | EG4 | SRF | 0.12 ± 0.11 | 8.3 | 29 | 0.24 ± 0.23 | 12.0 | 35 | 0.04 ± 0.02 | 1.1 | 23 | 0.14 ± 0.09 | 4.3 | 19 | 0.12 ± 0.17 | 4.3 | 15 | 0.14 ± 0.13 | 10.4 | 32 | 0.13 ± 0.10 | 2.2 | 21 |
| EGC | EG4 | EPI | 0.07 ± 0.05 | 1.4 | 33 | 0.47 ± 0.11 | 8.5 | 66 | 0.08 ± 0.04 | 1.5 | 31 | 0.11 ± 0.06 | 4.6 | 43 | 0.12 ± 0.08 | 4.9 | 34 | 0.24 ± 0.09 | 4.8 | 41 | 0.13 ± 0.08 | 2.3 | 44 |
| EGC | EG4 | MESO | 0.02 ± 0.01 | 1.5 | 33 | 0.01 ± 0.01 | 1.1 | 19 | 0.02 ± 0.01 | 2.2 | 29 | 0.01 ± 0.00 | 1.0 | 19 | 0.04 ± 0.03 | 3.9 | 18 | 0.01 ± 0.00 | 1.0 | 22 | 0.01 ± 0.01 | 1.5 | 24 |
| EGC | EG4 | BATHY | 0.01 ± 0.00 | 1.9 | 11 | 0.01 ± 0.01 | 1.1 | 25 | 0.02 ± 0.01 | 3.9 | 18 | 0.01 ± 0.01 | 2.1 | 14 | 0.02 ± 0.01 | 3.3 | 33 | 0.01 ± 0.00 | 1.6 | 5 | 0.01 ± 0.01 | 3.2 | 11 |
| WSC | HG1 | SRF | 0.14 ± 0.08 | 0.5 | 22 | 2.71 ± 0.84 | 8.6 | 20 | 0.10 ± 0.06 | 0.4 | 26 | 0.14 ± 0.09 | 0.6 | 37 | 0.11 ± 0.05 | 0.4 | 16 | 1.09 ± 0.34 | 3.8 | 23 | 1.18 ± 0.32 | 3.8 | 19 |
| WSC | HG1 | EPI | 0.08 ±  0.05 | 1.0 | 34 | 0.16 ± 0.08 | 2.4 | 35 | 0.05 ± 0.02 | 0.6 | 30 | 0.41 ± 0.14 | 6.2 | 51 | 0.31 ± 0.22 | 4.2 | 35 | 0.11 ± 0.07 | 1.6 | 37 | 0.17 ± 0.07 | 2.1 | 42 |
| WSC | HG1 | MESO | 0.02 ± 0.01 | 1.4 | 19 | 0.02 ± 0.01 | 1.3 | 20 | 0.03 ± 0.02 | 2.2 | 40 | 0.02 ± 0.02 | 1.6 | 33 | 0.06 ± 0.05 | 3.5 | 28 | 0.03 ± 0.01 | 2.4 | 24 | 0.02 ± 0.02 | 1.7 | 33 |
| WSC | HG1 | BATHY | 0.02 ± 0.01 | 2.9 | 27 | 0.02 ± 0.01 | 1.8 | 11 | 0.02 ± 0.01 | 2.4 | 33 | 0.01 ± 0.01 | 1.9 | 21 | 0.11 ± 0.09 | 4.9 | 26 | 0.02 ± 0.01 | 2.5 | 27 | 0.03 ± 0.02 | 3.6 | 23 |
| WSC | HG2 | SRF | 0.83 ± 0.52 | 4.0 | 32 | 4.82 ± 0.57 | 21.1 | 29 | 0.97 ± 0.58 | 4.6 | 24 | 0.08 ± 0.05 | 0.4 | 32 | 0.08 ± 0.05 | 0.4 | 25 | 2.84 ± 0.52 | 13.6 | 32 | 2.06 ± 0.45 | 10.0 | 29 |
| WSC | HG2 | EPI | 0.09 ± 0.05 | 1.2 | 33 | 0.32 ± 0.14 | 5.6 | 28 | 0.15 ± 0.14 | 2.1 | 32 | 0.41 ± 0.14 | 6.1 | 40 | 0.26 ± 0.26 | 3.3 | 24 | 0.20 ± 0.08 | 3.0 | 40 | 0.17 ± 0.08 | 2.5 | 38 |
| WSC | HG2 | MESO | 0.01 ± 0.01 | 1.7 | 17 | 0.02 ± 0.01 | 2.4 | 15 | 0.03 ± 0.02 | 3.8 | 54 | 0.01 ± 0.01 | 1.9 | 16 | 0.06 ± 0.04 | 5.0 | 17 | 0.02 ± 0.01 | 2.6 | 29 | 0.02 ± 0.01 | 2.2 | 22 |
| WSC | HG2 | BATHY | 0.02 ± 0.01 | 2.2 | 19 | 0.01 ± 0.00 | 1.6 | 11 | 0.03 ± 0.02 | 2.9 | 42 | 0.01 ± 0.01 | 1.8 | 13 | 0.11 ± 0.05 | 10.3 | 21 | 0.02 ± 0.02 | 3.3 | 23 | 0.04 ± 0.03 | 4.4 | 22 |
| WSC | HG4 | SRF | 0.25 ± 0.33 | 5.0 | 44 | 1.23 ± 0.30 | 22.5 | 31 | 0.36 ± 0.20 | 7.9 | 32 | 0.07 ± 0.04 | 0.6 | 28 | 0.29 ± 0.17 | 3.3 | 26 | 0.98 ± 0.29 | 19.6 | 45 | 0.11 ± 0.06 | 1.9 | 44 |
| WSC | HG4 | EPI | 0.18 ± 0.14 | 3.3 | 38 | 0.22 ± 0.13 | 5.2 | 34 | 0.20 ± 0.17 | 5.2 | 36 | 0.41 ± 0.10 | 7.0 | 42 | 0.45 ± 0.20 | 7.1 | 41 | 0.22 ± 0.13 | 4.1 | 39 | 0.08 ± 0.05 | 1.3 | 39 |
| WSC | HG4 | MESO | 0.01 ± 0.01 | 2.0 | 20 | 0.02 ± 0.01 | 1.4 | 17 | 0.06 ± 0.05 | 4.5 | 24 | 0.01 ± 0.00 | 1.1 | 24 | 0.06 ± 0.04 | 4.6 | 48 | 0.02 ± 0.01 | 2.4 | 17 | 0.02 ± 0.02 | 1.5 | 24 |
| WSC | HG4 | BATHY | 0.02 ± 0.01 | 3.6 | 17 | 0.01 ± 0.00 | 2.2 | 5 | 0.02 ± 0.01 | 4.2 | 32 | 0.01 ± 0.01 | 0.9 | 4 | 0.06 ± 0.05 | 8.2 | 12 | 0.01 ± 0.00 | 2.1 | 8 | 0.02 ± 0.01 | 3.9 | 20 |
| WSC | HG5 | SRF | 0.15 ± 0.08 | 1.2 | 26 | 0.49 ± 0.40 | 8.9 | 20 | 0.02 ± 0.01 | 0.5 | 14 | 0.04 ± 0.02 | 0.3 | 26 | 0.05 ± 0.04 | 0.5 | 14 | 1.49 ± 0.50 | 11.6 | 26 | 0.54 ± 0.20 | 4.7 | 25 |
| WSC | HG5 | EPI | 0.03 ± 0.01 | 0.5 | 38 | 0.10 ± 0.07 | 2.1 | 29 | 0.03 ± 0.02 | 0.7 | 27 | 0.03 ± 0.02 | 1.4 | 17 | 0.07 ± 0.04 | 3.7 | 37 | 0.11 ± 0.05 | 2.2 | 46 | 0.05 ± 0.02 | 1.1 | 42 |
| WSC | HG5 | MESO | 0.02 ± 0.00 | 5.9 | 2 | 0.01 ± 0.00 | 2.0 | 4 | 0.01 ± 0.01 | 3.3 | 23 | 0.02 ± 0.00 | 3.2 | 2 | 0.03 ± 0.02 | 9.6 | 14 | 0.01 ± 0.00 | 3.0 | 4 | 0.01 ± 0.01 | 3.7 | 4 |
| WSC | HG5 | BATHY | 0.01 ± 0.01 | 7.7 | 5 | 0.01 ± 0.00 | 4.6 | 11 | 0.01 ± 0.00 | 6.8 | 24 | 0.03 ± 0.01 | 12.1 | 5 | 0.02 ± 0.01 | 10.1 | 34 | 0.01 ± 0.00 | 5.0 | 4 | 0.01 ± 0.00 | 6.1 | 11 |
| WSC | HG7 | SRF | 0.22 ± 0.15 | 1.9 | 31 | 1.44 ± 0.72 | 12.3 | 29 | 0.03 ± 0.03 | 0.2 | 11 | 0.05 ± 0.03 | 0.4 | 36 | 0.04 ± 0.03 | 0.3 | 18 | 1.41 ± 0.36 | 12.2 | 31 | 0.66 ± 0.17 | 5.7 | 19 |
| WSC | HG7 | EPI | 0.05 ± 0.03 | 1.2 | 27 | 0.23 ± 0.09 | 5.1 | 32 | 0.04 ± 0.01 | 0.8 | 33 | 0.39 ± 0.10 | 6.1 | 53 | 0.13 ± 0.15 | 1.9 | 18 | 0.15 ± 0.06 | 3.4 | 30 | 0.07 ± 0.04 | 1.7 | 45 |
| WSC | HG7 | MESO | 0.02 ± 0.01 | 2.4 | 10 | 0.01 ± 0.01 | 1.5 | 14 | 0.02 ± 0.01 | 2.4 | 35 | 0.01 ± 0.00 | 5.6 | 3 | 0.02 ± 0.01 | 6.1 | 17 | 0.01 ± 0.00 | 1.6 | 19 | 0.01 ± 0.01 | 1.7 | 17 |
| WSC | HG7 | BATHY | 0.01 ± 0.01 | 2.7 | 16 | 0.01 ± 0.00 | 1.6 | 8 | 0.02 ± 0.01 | 5.1 | 24 | 0.01 ± 0.00 | 3.4 | 5 | 0.06 ± 0.04 | 12.0 | 18 | 0.02 ± 0.01 | 3.2 | 29 | 0.02 ± 0.01 | 8.0 | 10 |
| WSC | HG9 | SRF | 0.22 ± 0.24 | 2.2 | 31 | 2.22 ± 0.59 | 23.0 | 34 | 0.13 ± 0.10 | 1.3 | 20 | 0.05 ± 0.02 | 0.5 | 19 | 0.21 ± 0.21 | 2.4 | 22 | 2.08 ± 0.42 | 20.5 | 32 | 0.52 ± 0.15 | 5.4 | 36 |
| WSC | HG9 | EPI | 0.06 ± 0.03 | 0.7 | 16 | 0.37 ± 0.13 | 4.8 | 37 | 0.05 ± 0.02 | 0.6 | 25 | 0.30 ± 0.13 | 3.4 | 43 | 0.20 ± 0.17 | 2.2 | 41 | 0.24 ± 0.12 | 3.0 | 22 | 0.19 ± 0.08 | 2.0 | 32 |
| WSC | HG9 | MESO | 0.01 ± 0.01 | 2.9 | 22 | 0.01 ± NA | 2.0 | 1 | 0.02 ± 0.01 | 2.8 | 41 | 0.02 ± 0.01 | 2.5 | 8 | 0.07 ± 0.04 | 7.4 | 15 | 0.02 ± 0.01 | 3.0 | 15 | 0.02 ± 0.01 | 3.2 | 10 |
| WSC | HG9 | BATHY | 0.02 ± 0.01 | 5.3 | 18 | 0.01 ± 0.01 | 4.4 | 4 | 0.02 ± 0.01 | 5.2 | 44 | 0.01 ± 0.01 | 3.1 | 6 | 0.04 ± 0.02 | 8.0 | 19 | 0.01 ± 0.00 | 3.4 | 14 | 0.02 ± 0.01 | 5.4 | 24 |
| N | N3 | SRF | 0.19 ± 0.14 | 1.0 | 32 | 1.57 ± 0.36 | 8.1 | 28 | 0.28 ± 0.33 | 1.5 | 35 | 0.10 ± 0.06 | 0.7 | 34 | 0.32 ± 0.29 | 2.2 | 36 | 0.85 ± 0.22 | 4.6 | 32 | 1.02 ± 0.36 | 5.4 | 28 |
| N | N3 | EPI | 0.31 ± 0.13 | 3.0 | 34 | 0.58 ± 0.22 | 5.9 | 35 | 0.30 ± 0.26 | 3.2 | 38 | 0.27 ± 0.10 | 2.8 | 40 | 0.34 ± 0.28 | 3.0 | 42 | 0.55 ± 0.17 | 5.3 | 34 | 0.29 ± 0.12 | 2.9 | 39 |
| N | N3 | MESO | 0.01 ± 0.00 | 1.6 | 23 | 0.02 ± 0.01 | 2.2 | 12 | 0.03 ± 0.02 | 4.1 | 48 | 0.01 ± 0.01 | 2.0 | 6 | 0.04 ± 0.02 | 4.7 | 32 | 0.02 ± 0.01 | 2.8 | 30 | 0.02 ± 0.01 | 2.5 | 27 |
| N | N3 | BATHY | 0.01 ± 0.00 | 3.3 | 9 | 0.01 ± 0.00 | 3.3 | 4 | 0.02 ± 0.01 | 6.9 | 38 | 0.01 ± 0.00 | 3.3 | 14 | 0.03 ± 0.01 | 7.6 | 43 | 0.01 ± 0.01 | 3.5 | 12 | 0.02 ± 0.01 | 5.4 | 18 |
| N | N4 | SRF | 0.16 ± 0.09 | 1.0 | 23 | 3.71 ± 0.68 | 22.8 | 27 | 0.21 ± 0.19 | 1.6 | 29 | 0.06 ± 0.04 | 0.4 | 17 | 0.17 ± 0.10 | 0.9 | 26 | 2.26 ± 0.50 | 13.9 | 23 | 0.98 ± 0.23 | 5.6 | 37 |
| N | N4 | EPI | 0.06 ± 0.04 | 0.9 | 17 | 0.43 ± 0.20 | 7.1 | 34 | 0.13 ± 0.12 | 2.2 | 40 | 0.34 ± 0.10 | 5.2 | 39 | 0.17 ± 0.19 | 2.5 | 15 | 0.31 ± 0.14 | 5.3 | 35 | 0.13 ± 0.11 | 2.1 | 32 |
| N | N4 | MESO | 0.02 ± 0.01 | 1.6 | 24 | 0.02 ± 0.01 | 1.9 | 19 | 0.02 ± 0.01 | 2.3 | 29 | 0.01 ± 0.00 | 1.0 | 7 | 0.04 ± 0.03 | 4.5 | 34 | 0.02 ± 0.01 | 2.1 | 31 | 0.02 ± 0.01 | 2.4 | 24 |
| N | N4 | BATHY | 0.02 ± 0.01 | 5.7 | 28 | 0.01 ± 0.00 | 4.1 | 16 | 0.02 ± 0.01 | 5.3 | 44 | 0.02 ± 0.01 | 4.0 | 13 | 0.04 ± 0.02 | 9.0 | 37 | 0.02 ± 0.01 | 6.1 | 26 | 0.02 ± 0.01 | 5.2 | 21 |
| N | N5 | SRF | 0.07 ± 0.03 | 0.4 | 13 | 0.90 ± 0.43 | 5.6 | 30 | 0.07 ± 0.08 | 0.5 | 6 | 0.06 ± 0.03 | 0.4 | 14 | 0.14 ± 0.07 | 0.8 | 24 | 3.06 ± 0.61 | 19.8 | 23 | 0.75 ± 0.29 | 4.9 | 26 |
| N | N5 | EPI | 0.12 ± 0.07 | 1.6 | 36 | 0.30 ± 0.12 | 5.0 | 33 | 0.06 ± 0.03 | 0.8 | 29 | 0.31 ± 0.12 | 4.5 | 43 | 0.19 ± 0.16 | 2.4 | 37 | 0.24 ± 0.11 | 3.5 | 40 | 0.10 ± 0.05 | 1.4 | 36 |
| N | N5 | MESO | 0.02 ± 0.01 | 1.6 | 26 | 0.02 ± 0.01 | 1.9 | 20 | 0.02 ± 0.02 | 2.6 | 39 | 0.01 ± 0.01 | 1.3 | 15 | 0.04 ± 0.03 | 4.4 | 33 | 0.02 ± 0.01 | 2.2 | 32 | 0.02 ± 0.01 | 2.1 | 23 |
| N | N5 | BATHY | 0.02 ± 0.01 | 4.6 | 16 | 0.01 ± 0.00 | 2.6 | 6 | 0.02 ± 0.01 | 5.1 | 33 | 0.01 ± 0.01 | 3.2 | 12 | 0.03 ± 0.02 | 7.7 | 43 | 0.01 ± 0.01 | 3.6 | 17 | 0.01 ± 0.01 | 3.3 | 13 |
| **Region** | **Station** | **Water layer** | POL | % | ***n*** | ROS | **%** | ***n*** | SAR11 | **%** | ***n*** | SAR202 | **%** | ***n*** | SAR324 | **%** | ***n*** | SAR406 | **%** | ***n*** | VER | **%** | ***n*** |
| EGC | EG1 | SRF | 0.76 ± 0.18 | 17.9 | 31 | 0.14 ± 0.13 | 3.8 | 19 | 1.15 ± 0.22 | 34.9 | 28 | 0.07 ± 0.03 | 2.4 | 19 | 0.06 ± 0.04 | 1.6 | 22 | 0.03 ± 0.00 | 0.9 | 2 | 0.05 ± 0.02 | 1.5 | 23 |
| EGC | EG1 | EPI | 0.04 ± 0.02 | 2.4 | 19 | 0.16 ± 0.13 | 8.1 | 21 | 0.40 ± 0.10 | 22.5 | 21 | 0.05 ± 0.03 | 2.3 | 21 | 0.10 ± 0.06 | 3.7 | 23 | 0.05 ± 0.02 | 2.3 | 2 | 0.04 ± 0.02 | 2.0 | 21 |
| EGC | EG1 | MESO | 0.02 ± 0.01 | 4.3 | 17 | 0.07 ± 0.03 | 15.4 | 42 | 0.10 ± 0.03 | 23.3 | 25 | 0.02 ± 0.02 | 6.1 | 42 | 0.04 ± 0.02 | 7.2 | 18 | 0.01 ± 0.00 | 1.9 | 4 | 0.01 ± 0.01 | 3.2 | 14 |
| EGC | EG4 | SRF | 0.09 ± 0.11 | 4.4 | 31 | 0.30 ± 0.30 | 10.2 | 25 | 2.56 ± 1.16 | 64.3 | 25 | 0.05 ± 0.03 | 1.2 | 25 | 0.13 ± 0.06 | 3.1 | 22 | 0.03 ± 0.00 | 1.3 | 11 | 0.12 ± 0.10 | 3.5 | 18 |
| EGC | EG4 | EPI | 0.12 ± 0.07 | 2.1 | 33 | 0.51 ± 0.35 | 9.8 | 39 | 1.63 ± 0.26 | 29.8 | 29 | 0.08 ± 0.04 | 1.5 | 39 | 0.09 ± 0.06 | 2.4 | 16 | 0.03 ± 0.00 | 1.2 | 4 | 0.08 ± 0.05 | 1.5 | 42 |
| EGC | EG4 | MESO | 0.01 ± 0.01 | 1.3 | 30 | 0.06 ± 0.04 | 6.7 | 33 | 0.21 ± 0.05 | 20.5 | 24 | 0.02 ± 0.01 | 2.4 | 33 | 0.05 ± 0.02 | 5.6 | 42 | 0.01 ± 0.00 | 0.9 | 7 | 0.02 ± 0.01 | 1.8 | 23 |
| EGC | EG4 | BATHY | 0.02 ± 0.01 | 1.6 | 29 | 0.03 ± 0.03 | 5.1 | 14 | 0.07 ± 0.07 | 13.9 | 32 | 0.02 ± 0.02 | 5.4 | 14 | 0.03 ± 0.01 | 4.7 | 35 | 0.01 ± 0.01 | 2.7 | 6 | 0.01 ± 0.01 | 2.0 | 11 |
| WSC | HG1 | SRF | 0.56 ± 1.67 | 1.8 | 20 | 2.08 ± 0.51 | 6.9 | 21 | 14.21 ± 2.03 | 44.9 | 22 | 0.07 ± 0.04 | 0.2 | 21 | 0.13 ± 0.07 | 0.5 | 29 | 0.03 ± 0.00 | 0.1 | 8 | 1.13 ± 0.31 | 3.5 | 19 |
| WSC | HG1 | EPI | 0.06 ± 0.03 | 0.9 | 20 | 0.26 ± 0.13 | 3.3 | 46 | 2.34 ± 0.41 | 33.2 | 37 | 0.06 ± 0.03 | 0.9 | 46 | 0.11 ± 0.06 | 1.2 | 21 | 0.05 ± 0.02 | 0.6 | 17 | 0.07 ± 0.04 | 0.9 | 35 |
| WSC | HG1 | MESO | 0.02 ± 0.01 | 1.7 | 29 | 0.06 ± 0.05 | 4.2 | 37 | 0.25 ± 0.06 | 22.9 | 30 | 0.04 ± 0.02 | 3.4 | 37 | 0.07 ± 0.03 | 4.2 | 23 | 0.01 ± 0.01 | 0.8 | 3 | 0.02 ± 0.01 | 1.7 | 30 |
| WSC | HG1 | BATHY | 0.03 ± 0.02 | 3.5 | 28 | 0.05 ± 0.03 | 6.7 | 39 | 0.10 ± 0.04 | 12.0 | 36 | 0.04 ± 0.02 | 4.4 | 39 | 0.05 ± 0.02 | 6.9 | 18 | 0.01 ± 0.00 | 1.9 | 8 | 0.02 ± 0.01 | 2.8 | 18 |
| WSC | HG2 | SRF | 1.16 ± 0.24 | 5.1 | 29 | 0.66 ± 0.11 | 2.9 | 32 | 9.78 ± 1.50 | 45.6 | 26 | 0.06 ± 0.04 | 0.3 | 32 | 0.08 ± 0.04 | 0.4 | 31 | 0.04 ± 0.02 | 0.2 | 11 | 2.02 ± 0.43 | 9.7 | 29 |
| WSC | HG2 | EPI | 0.07 ± 0.04 | 1.2 | 27 | 0.17 ± 0.13 | 2.5 | 38 | 2.48 ± 0.45 | 31.5 | 23 | 0.04 ± 0.02 | 0.6 | 38 | 0.08 ± 0.04 | 1.0 | 30 | 0.04 ± 0.01 | 0.5 | 12 | 0.16 ± 0.07 | 2.4 | 37 |
| WSC | HG2 | MESO | 0.01 ± 0.01 | 2.4 | 20 | 0.10 ± 0.04 | 12.5 | 37 | 0.17 ± 0.05 | 18.2 | 29 | 0.03 ± 0.01 | 3.5 | 37 | 0.06 ± 0.02 | 6.4 | 25 | 0.01 ± 0.00 | 1.6 | 8 | 0.02 ± 0.01 | 2.4 | 25 |
| WSC | HG2 | BATHY | 0.02 ± 0.01 | 3.0 | 27 | 0.06 ± 0.02 | 8.0 | 35 | 0.13 ± 0.04 | 16.6 | 34 | 0.03 ± 0.02 | 4.5 | 35 | 0.05 ± 0.03 | 6.1 | 25 | 0.01 ± 0.00 | 1.7 | 13 | 0.02 ± 0.01 | 2.6 | 19 |
| WSC | HG4 | SRF | 0.50 ± 0.21 | 9.2 | 31 | 0.23 ± 0.32 | 4.3 | 27 | 1.54 ± 0.36 | 26.9 | 25 | 0.04 ± 0.01 | 0.8 | 27 | 0.05 ± 0.03 | 0.7 | 16 | 0.05 ± 0.02 | 1.4 | 5 | 0.09 ± 0.05 | 1.5 | 43 |
| WSC | HG4 | EPI | 0.08 ± 0.05 | 1.9 | 29 | 0.09 ± 0.03 | 2.1 | 37 | 2.29 ± 0.33 | 51.2 | 37 | 0.06 ± 0.03 | 1.4 | 37 | 0.10 ± 0.08 | 1.7 | 54 | 0.04 ± 0.01 | 0.7 | 10 | 0.06 ± 0.03 | 0.9 | 28 |
| WSC | HG4 | MESO | 0.02 ± 0.01 | 1.4 | 27 | 0.09 ± 0.05 | 7.8 | 39 | 0.22 ± 0.06 | 21.4 | 22 | 0.05 ± 0.04 | 4.1 | 39 | 0.05 ± 0.02 | 4.7 | 32 | 0.02 ± 0.01 | 1.4 | 15 | 0.02 ± 0.02 | 1.9 | 25 |
| WSC | HG4 | BATHY | 0.02 ± 0.01 | 3.3 | 21 | 0.03 ± 0.02 | 5.9 | 35 | 0.08 ± 0.03 | 17.0 | 35 | 0.03 ± 0.02 | 4.5 | 35 | 0.04 ± 0.04 | 6.2 | 10 | 0.02 ± 0.01 | 1.9 | 2 | 0.01 ± 0.01 | 3.0 | 13 |
| WSC | HG5 | SRF | 0.21 ± 0.19 | 3.7 | 19 | 0.37 ± 0.16 | 2.9 | 34 | 2.66 ± 1.41 | 40.4 | 25 | 0.03 ± 0.03 | 0.7 | 34 | 0.03 ± 0.02 | 0.5 | 22 | 0.03 ± 0.02 | 0.2 | 17 | 1.28 ± 0.45 | 4.7 | 25 |
| WSC | HG5 | EPI | 0.05 ± 0.03 | 1.1 | 31 | 0.08 ± 0.05 | 2.1 | 25 | 1.25 ± 0.43 | 25.8 | 29 | 0.04 ± 0.03 | 0.8 | 25 | 0.03 ± 0.03 | 1.5 | 18 | 0.01 ± 0.00 | 0.8 | 3 | 0.14 ± 0.06 | 1.2 | 42 |
| WSC | HG5 | MESO | 0.01 ± 0.00 | 2.8 | 16 | 0.02 ± 0.01 | 4.4 | 19 | 0.04 ± 0.02 | 12.7 | 27 | 0.02 ± 0.01 | 7.4 | 19 | 0.01 ± 0.01 | 4.5 | 18 | 0.01 ± NA | 2.0 | 1 | 0.01 ± 0.00 | 3.8 | 3 |
| WSC | HG5 | BATHY | 0.01 ± 0.01 | 5.5 | 16 | 0.03 ± 0.02 | 18.7 | 42 | 0.05 ± 0.02 | 31.4 | 38 | 0.01 ± 0.01 | 8.5 | 42 | 0.02 ± 0.01 | 6.0 | 25 | 0.02 ± NA | 5.9 | 1 | 0.01 ± 0.00 | 5.7 | 5 |
| WSC | HG7 | SRF | 0.82 ± 0.47 | 7.0 | 29 | 0.31 ± 0.07 | 2.5 | 24 | 4.66 ± 0.99 | 38.6 | 30 | 0.04 ± 0.05 | 0.3 | 24 | 0.04 ± 0.02 | 0.3 | 25 | 0.02 ± 0.01 | 0.2 | 20 | 1.53 ± 0.40 | 5.6 | 19 |
| WSC | HG7 | EPI | 0.08 ± 0.04 | 1.7 | 32 | 0.09 ± 0.04 | 2.0 | 38 | 1.30 ± 0.26 | 31.1 | 28 | 0.04 ± 0.02 | 1.0 | 38 | 0.09 ± 0.08 | 2.3 | 3 | 0.02 ± 0.01 | 0.8 | 11 | 0.14 ± 0.09 | 1.4 | 45 |
| WSC | HG7 | MESO | 0.01 ± 0.01 | 1.8 | 21 | 0.04 ± 0.02 | 5.3 | 36 | 0.15 ± 0.04 | 18.2 | 23 | 0.03 ± 0.02 | 4.0 | 36 | 0.03 ± 0.02 | 6.5 | 32 | 0.01 ± 0.00 | 3.7 | 3 | 0.02 ± 0.01 | 2.7 | 8 |
| WSC | HG7 | BATHY | 0.03 ± 0.02 | 5.9 | 21 | 0.04 ± 0.03 | 27.9 | 23 | 0.12 ± 0.04 | 26.2 | 25 | 0.04 ± 0.03 | 13.1 | 23 | 0.03 ± 0.02 | 5.8 | 35 | 0.01 ± 0.01 | 4.3 | 9 | 0.01 ± 0.01 | 4.2 | 8 |
| WSC | HG9 | SRF | 1.26 ± 0.31 | 13.1 | 34 | 0.29 ± 0.13 | 2.9 | 27 | 3.53 ± 0.79 | 33.2 | 22 | 0.06 ± 0.06 | 0.6 | 27 | 0.07 ± 0.04 | 0.7 | 23 | 0.04 ± 0.02 | 0.5 | 10 | 0.53 ± 0.14 | 5.2 | 36 |
| WSC | HG9 | EPI | 0.11 ± 0.05 | 1.4 | 37 | 0.20 ± 0.11 | 2.4 | 39 | 1.78 ± 0.49 | 19.9 | 21 | 0.06 ± 0.05 | 0.7 | 39 | 0.10 ± 0.04 | 1.0 | 15 | 0.04 ± 0.02 | 0.6 | 11 | 0.18 ± 0.08 | 1.9 | 32 |
| WSC | HG9 | MESO | 0.02 ± 0.01 | 3.1 | 15 | 0.03 ± 0.02 | 6.1 | 27 | 0.11 ± 0.04 | 19.6 | 35 | 0.03 ± 0.01 | 4.4 | 27 | 0.05 ± 0.02 | 6.0 | 38 | 0.02 ± 0.02 | 3.3 | 7 | 0.02 ± 0.01 | 3.1 | 7 |
| WSC | HG9 | BATHY | 0.02 ± 0.01 | 6.6 | 16 | 0.05 ± 0.03 | 15.8 | 23 | 0.09 ± 0.03 | 25.5 | 33 | 0.02 ± 0.01 | 7.7 | 23 | 0.03 ± 0.01 | 6.2 | 25 | 0.01 ± 0.00 | 2.7 | 12 | 0.01 ± 0.01 | 4.3 | 11 |
| N | N3 | SRF | 0.29 ± 0.08 | 1.5 | 28 | 0.61 ± 0.14 | 3.2 | 29 | 6.10 ± 0.60 | 32.1 | 33 | 0.07 ± 0.06 | 0.4 | 29 | 0.08 ± 0.04 | 0.5 | 32 | 0.08 ± 0.05 | 0.4 | 24 | 1.01 ± 0.37 | 5.3 | 28 |
| N | N3 | EPI | 0.11 ± 0.05 | 1.1 | 33 | 0.22 ± 0.09 | 2.0 | 51 | 2.71 ± 0.44 | 28.0 | 27 | 0.04 ± 0.02 | 0.4 | 51 | 0.07 ± 0.04 | 0.6 | 37 | 0.04 ± 0.02 | 0.4 | 13 | 0.28 ± 0.12 | 2.8 | 39 |
| N | N3 | MESO | 0.02 ± 0.01 | 2.0 | 19 | 0.12 ± 0.05 | 17.2 | 39 | 0.14 ± 0.05 | 23.1 | 41 | 0.03 ± 0.02 | 4.4 | 39 | 0.05 ± 0.02 | 6.4 | 16 | 0.01 ± 0.00 | 1.4 | 3 | 0.02 ± 0.01 | 2.9 | 29 |
| N | N3 | BATHY | 0.02 ± 0.01 | 7.3 | 18 | 0.06 ± 0.02 | 17.9 | 44 | 0.07 ± 0.03 | 23.9 | 31 | 0.03 ± 0.02 | 9.6 | 44 | 0.03 ± 0.02 | 6.9 | 7 | 0.02 ± 0.01 | 4.7 | 11 | 0.01 ± 0.01 | 3.6 | 16 |
| N | N4 | SRF | 1.26 ± 0.22 | 7.8 | 27 | 0.62 ± 0.17 | 3.4 | 32 | 3.93 ± 0.71 | 22.8 | 24 | 0.09 ± 0.06 | 0.7 | 32 | 0.07 ± 0.04 | 0.4 | 30 | 0.04 ± 0.02 | 0.2 | 12 | 0.97 ± 0.23 | 5.4 | 37 |
| N | N4 | EPI | 0.13 ± 0.05 | 2.0 | 32 | 0.25 ± 0.10 | 3.6 | 31 | 1.64 ± 0.30 | 23.6 | 22 | 0.06 ± 0.03 | 1.0 | 31 | 0.06 ± 0.03 | 0.9 | 27 | 0.04 ± 0.01 | 0.5 | 9 | 0.10 ± 0.06 | 1.8 | 29 |
| N | N4 | MESO | 0.03 ± 0.02 | 2.8 | 26 | 0.10 ± 0.06 | 9.8 | 41 | 0.18 ± 0.05 | 18.0 | 21 | 0.03 ± 0.02 | 3.1 | 41 | 0.06 ± 0.02 | 5.4 | 24 | 0.01 ± 0.00 | 1.2 | 8 | 0.02 ± 0.01 | 1.9 | 24 |
| N | N4 | BATHY | 0.02 ± 0.01 | 5.2 | 23 | 0.07 ± 0.04 | 22.4 | 34 | 0.12 ± 0.05 | 34.0 | 22 | 0.02 ± 0.01 | 7.1 | 34 | 0.05 ± 0.03 | 9.6 | 16 | 0.01 ± 0.01 | 2.9 | 19 | 0.01 ± 0.00 | 4.2 | 14 |
| N | N5 | SRF | 2.48 ± 0.40 | 15.5 | 30 | 0.48 ± 0.13 | 2.9 | 31 | 2.54 ± 0.46 | 15.6 | 24 | 0.05 ± 0.03 | 0.3 | 31 | 0.06 ± 0.04 | 0.4 | 18 | 0.04 ± 0.02 | 0.3 | 11 | 0.84 ± 0.24 | 4.9 | 26 |
| N | N5 | EPI | 0.10 ± 0.05 | 1.6 | 33 | 0.18 ± 0.17 | 2.5 | 26 | 2.39 ± 0.64 | 35.5 | 24 | 0.05 ± 0.02 | 0.7 | 26 | 0.08 ± 0.04 | 1.1 | 33 | 0.03 ± 0.00 | 0.5 | 6 | 0.08 ± 0.04 | 1.2 | 30 |
| N | N5 | MESO | 0.02 ± 0.01 | 1.9 | 25 | 0.13 ± 0.05 | 12.9 | 48 | 0.20 ± 0.06 | 19.6 | 39 | 0.02 ± 0.01 | 1.9 | 48 | 0.06 ± 0.03 | 6.0 | 26 | 0.01 ± 0.00 | 1.1 | 7 | 0.02 ± 0.01 | 2.0 | 20 |
| N | N5 | BATHY | 0.02 ± 0.01 | 3.6 | 14 | 0.07 ± 0.03 | 19.9 | 44 | 0.07 ± 0.02 | 21.6 | 32 | 0.03 ± 0.01 | 6.5 | 44 | 0.03 ± 0.01 | 6.1 | 31 | 0.01 ± 0.00 | 2.8 | 7 | 0.02 ± 0.01 | 4.7 | 18 |

**Table S4**. Pearson’s correlation coefficient (*r*) tests of investigated bacterioplankton groups with environmental parameters (n=10), and phytoplankton cell abundances (n=5) in surface waters of the Fram Strait. Combinations that show significant correlation are marked with grey shadow. *Bacteria* (EUB)*, Archaea* (ARCH), *Alteromonadaceae/Colwelliaceae/Pseudoalteromonadaceae* (ATL), *Bacteroidetes* (BACT), *Chloroflexi* (CFX)*, Thaumarchaeota* (THA), *Deltaproteobacteria* (DELTA)*, Gammaproteobacteria* (GAM), *Opitutales* (OPI)*, Polaribacter* (POL)*, Rhodobacteraceae* (ROS), *Verrucomicrobia* (VER), SAR202, SAR324, SAR406 and SAR11 clades.

| **Taxa** | **Temperature** | | **Salinity** | | **Chlorophyll *a* conc.** | | **△NO_3_** | | **△PO_4_** | | **△SO_3_** | | **Diatoms** | | ***Phaeocystis spp.*** | |
| --- | --- | --- | --- | --- | --- | --- | --- | --- | --- | --- | --- | --- | --- | --- | --- | --- |
|  | ***r*** | ***p-value*** | ***r*** | ***p-value*** | ***r*** | ***p-value*** | ***r*** | ***p-value*** | ***r*** | ***p-value*** | ***r*** | ***p-value*** | ***r*** | ***p-value*** | ***r*** | ***p-value*** |
| ALT | 0.03 | 0.93 | 0.28 | 0.41 | -0.28 | 0.40 | -0.12 | 0.74 | 0.27 | 0.44 | -0.23 | 0.519 | 0.49 | 0.40 | -0.84 | 0.08 |
| ARCH | 0.47 | 0.14 | 0.36 | 0.28 | -0.50 | 0.12 | -0.31 | 0.39 | -0.21 | 0.56 | -0.63 | 0.05 | 0.87 | 0.06 | -0.15 | 0.81 |
| BACT | 0.41 | 0.21 | 0.52 | 0.10 | -0.25 | 0.46 | 0.14 | 0.70 | 0.56 | 0.09 | 0.01 | 0.98 | 0.40 | 0.50 | -0.36 | 0.55 |
| CFX | 0.12 | 0.72 | 0.40 | 0.23 | -0.25 | 0.45 | 0.07 | 0.85 | 0.45 | 0.19 | -0.12 | 0.74 | -0.22 | 0.72 | -0.38 | 0.53 |
| THA | 0.35 | 0.30 | 0.59 | 0.06 | -0.30 | 0.37 | -0.1 | 0.79 | -0.09 | 0.80 | -0.05 | 0.90 | -0.19 | 0.76 | 0.78 | 0.12 |
| DELTA | -0.4 | 0.23 | 0.05 | 0.89 | -0.43 | 0.18 | 0.04 | 0.91 | -0.15 | 0.68 | -0.60 | 0.07 | 0.38 | 0.53 | **-0.89** | **0.04** |
| EUB | 0.60 | 0.05 | 0.50 | 0.12 | 0.22 | 0.52 | 0.17 | 0.64 | 0.59 | 0.07 | 0.14 | 0.69 | -0.32 | 0.60 | 0.19 | 0.76 |
| GAM | 0.14 | 0.69 | 0.04 | 0.91 | 0.50 | 0.12 | 0.42 | 0.22 | **0.74** | **0.01** | 0.41 | 0.25 | -0.34 | 0.57 | 0.08 | 0.90 |
| OPI | **0.61** | **0.05** | 0.38 | 0.25 | -0.02 | 0.94 | 0.06 | 0.87 | 0.49 | 0.15 | -0.02 | 0.96 | -0.01 | 0.98 | 0.11 | 0.85 |
| POL | -0.10 | 0.78 | 0.00 | 0.99 | 0.59 | 0.05 | 0.33 | 0.36 | 0.54 | 0.12 | 0.25 | 0.48 | -0.30 | 0.62 | 0.03 | 0.96 |
| ROS | **0.78** | **0.00** | 0.49 | 0.13 | 0.06 | 0.87 | 0.26 | 0.47 | 0.54 | 0.12 | 0.09 | 0.80 | -0.13 | 0.84 | 0.32 | 0.60 |
| SAR11 | **0.79** | **0.00** | **0.61** | **0.04** | -0.09 | 0.78 | -0.15 | 0.69 | 0.30 | 0.40 | -0.08 | 0.82 | 0.02 | 0.97 | 0.43 | 0.47 |
| SAR202 | 0.16 | 0.64 | 0.29 | 0.39 | -0.51 | 0.11 | 0.23 | 0.51 | 0.33 | 0.36 | -0.37 | 0.29 | 0.82 | 0.09 | -0.33 | 0.59 |
| SAR324 | 0.14 | 0.69 | **0.64** | **0.03** | -0.39 | 0.24 | 0.21 | 0.56 | 0.22 | 0.55 | 0.03 | 0.93 | 0.02 | 0.97 | 0.77 | 0.13 |
| SAR406 | 0.04 | 0.92 | 0.26 | 0.44 | -0.33 | 0.32 | 0.18 | 0.62 | 0.18 | 0.63 | -0.29 | 0.41 | -0.58 | 0.30 | -0.18 | 0.77 |
| VER | **0.68** | **0.02** | 0.06 | 0.86 | 0.20 | 0.55 | -0.18 | 0.61 | 0.20 | 0.59 | 0.10 | 0.78 | -0.10 | 0.87 | 0.17 | 0.78 |

**Figure S1.** Proportion abundance of the targeted taxonomic groups in CARD-FISH and in high-throughput 16S rRNA gene sequencing performed during PS99.2. The different geographical regions of the Fram Strait are indicated by color: ice-covered EGC – blue, ice-margin N – gray, ice-free WSC – red.

* *Alteromonadaceae/Colwelliaceae/Pseudoalteromonadaceae.*

**Figure S2.** Pearsons’s correlation coefficient (*r*) plot between environmental parameters representing the distinct water masses (temperature and salinity) and the different phytoplankton bloom conditions (chlorophyll *a* concentration and consumed inorganic nutrients) across the Fram Strait. Insignificant correlations are crossed with (X).
